# Curcumin and neuroplasticity: epigenetic mechanisms underlying cognitive enhancement in aging and neurodegenerative disorders

**DOI:** 10.3389/fnagi.2025.1592280

**Published:** 2025-08-07

**Authors:** Hao Jiao, Xiuying Wang, Dahui Zhang, Shengxue Zhou, Feng Gao

**Affiliations:** ^1^College of Chinese Medicine, Jilin Agricultural Science and Technology College, Jilin, China; ^2^Dehui Employment Training Center, Dehui, Jilin, China

**Keywords:** curcumin, DNA acetylation, non-coding RNAs, epigenetic, neurodegenerative disorders

## Abstract

Aging and neurodegenerative diseases are characterized by cognitive decline, impaired neuroplasticity, and epigenetic dysregulation. Curcumin, a bioactive polyphenol derived from *Curcuma longa*, has gained significant attention for its neuroprotective properties, particularly in enhancing cognitive function through epigenetic mechanisms. This review explores the multifaceted role of curcumin in modulating key molecular pathways involved in neuroplasticity, including histone modifications, DNA methylation, and non-coding RNA regulation. Additionally, curcumin influences neurogenesis, synaptic remodeling, and mitochondrial biogenesis, which are critical for maintaining brain function in aging and neurodegenerative conditions such as Alzheimer’s and Parkinson’s disease. By targeting neuroinflammatory and oxidative stress pathways, curcumin further supports cognitive resilience and neuronal survival. We also discuss the therapeutic implications of curcumin as a potential epigenetic modulator and neurogenic agent, emphasizing its synergistic effects with lifestyle interventions such as physical activity and dietary strategies. Despite promising preclinical and clinical findings, challenges related to curcumin’s bioavailability and translational efficacy remain. Future research should focus on optimizing delivery systems and exploring combination therapies to enhance curcumin’s neuroprotective benefits. This review highlights curcumin as a promising candidate for promoting cognitive longevity and mitigating neurodegeneration through epigenetic reprogramming.

## 1 Introduction

The steady rise in global life expectancy has prompted extensive research into the biological mechanisms of aging. This research focuses on identifying and understanding the biochemical and genetic processes responsible for age-related decline. Recent findings suggest that aging is not an immutable biological reality but rather a complex process that can be manipulated and modulated. Aging is an irreversible physiological process characterized by the development of various age-related diseases, including osteoporosis, diabetes, arthritis, cerebrovascular disease, musculoskeletal disorders, neurodegenerative conditions, cardiovascular disease, cancer, cataracts, and atherosclerosis (Li Z. et al., 2021). However, it has been demonstrated that the rate and quality of aging can be influenced by specific interventions. Hallmarks of aging include genetic instability, epigenetic alterations, telomere shortening, cellular senescence, stem cell exhaustion, and mitochondrial dysfunction (López-Otín et al., 2013). Indeed, aging is associated with a multitude of epigenetic alterations observed across various species. These epigenetic modifications contribute directly to the aging process and age-related pathologies. Specifically, these changes encompass the accumulation of histone variants, alterations in chromatin accessibility, histone loss and heterochromatin reduction, aberrant histone modifications, and dysregulated expression and activity of non-coding RNAs. These epigenetic modifications impact cellular processes, ultimately leading to the onset and progression of various human diseases as mentioned above (Saul and Kosinsky, 2021).

Diet plays a crucial role in mitigating the effects of aging. Consumption of foods rich in antioxidants and anti-inflammatory compounds such as polyphenols has been associated with a reduced risk of age-related cognitive impairment and neurodegenerative diseases (Izadi et al., 2024; Zhu et al., 2025). Polyphenols could modulate epigenetic mechanisms such as DNA methylation. Qin and colleagues showed DNMT1 and DNMT3b inhibition following treatment of breast cancer cells with resveratrol in a dose dependent manner (Qin et al., 2005). Or in another study, EGCG inhibited different DNMTs especially DNMT3A in different cancer cell lines (Fang et al., 2003). Naponelli et al. (2017) also showed the inhibitory role of EGCG on DNMTs in prostate cancer cell lines. Thus, different polyphenols could modulate DNA methylations via affecting different DNMTs. Among nutrients that are helpful against aging, recent research has highlighted the therapeutic potential of curcumin. Curcumin, 1,7-bis [4-hydroxy 3-methoxy phenyl]-1,6-heptadiene-3,5-dione, is a polyphenol compound that is found in the rhizome of *Curcuma longa* Linn (Li Z. et al., 2021). Besides the culinary applications of curcumin, it is widely used as a beneficial compound in the field of medicine which can provide a variety of preventive or therapeutic roles against different diseases (López-Otín et al., 2013; Saul and Kosinsky, 2021; Izadi et al., 2024). For instance, it serves as an anti-inflammatory agent that is used in non-alcoholic fatty liver disease, endometriosis, and neurodegenerative diseases along with a variety of several other diseases (Vallée and Lecarpentier, 2020; Saadati et al., 2019; Azzini et al., 2024). Furthermore, it functions as an anti-aging, anti-oxidative, epigenetic modulator, antimicrobial, anti-angiogenic, and anti-mutagenic agent (Izadi et al., 2024; Jakubczyk et al., 2020; Hassan et al., 2019; Kocaadam and Şanlier, 2017; Menon and Sudheer, 2007; Adibian et al., 2019; Alizadeh et al., 2018; Asadi et al., 2019; Ms et al., 2020). Recent studies have indicated that curcumin is capable of serving as an inhibitor of histone acetyltransferase and DNMTs while modulating the methylation level of DNA regions and regulating various non-coding RNAs (i.e., microRNA and lncRNA) (Amini et al., 2021; Reuter et al., 2011; Kumar et al., 2024). Furthermore, studies have shown that curcumin not only could mitigate age-related diseases such as cancer but also improves the life span through different mechanisms such as improvement of telomere stability as well as modulation of age-related signaling pathways which discussed elsewhere (Zia et al., 2021). Therefore, we aim to investigate the role of curcumin as an epigenetic modulator in age-related diseases, such as cardiovascular diseases, diabetes, osteoporosis, cataracts, dementias, and stroke, and neurodegenerative disorders.

## 2 Curcumin

### 2.1 Chemistry

Turmeric is chemically composed of a variety of components which include carbohydrates, protein, fat, vitamins, and moisture. Furthermore, it contains essential oils (i.e., phellandrene, sabinene, cineol, borneol, zingiberene, and sesquiterpenes), minerals (i.e., iron, potassium, sodium, calcium, and phosphorus), and curcuminoids (Strimpakos and Sharma, 2008; Priyadarsini, 2014; Nelson et al., 2017; Prasad et al., 2014; Goel et al., 2008). Approximately 77% of its curcuminoids is curcumin, while demethoxyxurcumin and bisdemethoxycurcumin make up 17% and 3–6%, respectively (Prasad et al., 2014; Anand et al., 2007). The elucidation of the structure of curcumin as diferuloylmethane or 1,6-heptadiene-3,5-dione-1,7-bis (4-hydroxy-3-methoxyphenyl)-(1E,6E) was reported by Melabedzka et al. in 1910 (Vogel and Pelletier, 1815). Curcumin exists in two tautomeric forms, keto, and enol, with its solubility being largely insoluble at room temperature in aqueous solutions under neutral and acidic pH conditions. However, curcumin dissolves in organic solvents like methanol, ethanol, and acetone due to its lipophilic nature with a log *p* value of approximately 3.0. While the keto form predominates in neutral and acidic pH, the enol tautomer is exclusively present in alkaline conditions (Priyadarsini, 2014; Priyadarsini, 2009; Payton et al., 2007). The solubility of curcumin in aqueous solutions increases under alkaline conditions but degrades rapidly in both neutral and alkaline environments (Goel et al., 2008; Aggarwal et al., 2003).

Curcumin possesses three reactive sites, namely a hydrogen atom donor, a Michael acceptor, and a metal chelator. Curcumin’s α,β-unsaturated β-diketone section serves as an effective metal-chelating agent, forming complexes with various metal ions. The metal-chelating capability of curcumin has been used as a therapeutic agent in different diseases, such as cancer, depression, and arthritis (Banerjee and Chakravarty, 2015; Mei et al., 2011; Pucci et al., 2012; Wanninger et al., 2015). By forming complexes with metals like Al^3+^, curcumin is helpful in Alzheimer’s disease (AD). On the other hand, it directly binds to small β-amyloid species to prevent aggregation and fibril formation. Additionally, through creating stable complexes with heavy metals like copper (Cu), chromium (Cr), arsenic (As), mercury (Hg), lead (Pb), and cadmium (Cd), curcumin mitigates the toxicity induced by heavy metal (García-Niño and Pedraza-Chaverrí, 2014; Yadav et al., 2012; Shukla et al., 2003; Agarwal et al., 2010; Flora et al., 2012; Oguzturk et al., 2012).

### 2.2 Metabolism

The liver is considered the primary organ responsible for curcumin metabolism (Garcea et al., 2004; Hoehle et al., 2006). In humans, UDP-glucuronosyltransferase (UGT) 1A1, 1A8, and 1A10 isoenzymes predominantly mediate glucuronidation in the intestinal epithelium. However, hepatic glucuronidation of curcumin is entirely facilitated by UGT1A1 (Hoehle et al., 2007). Interestingly, the intestinal epithelium exhibits a higher binding rate of curcumin compared to liver microsomes, which contrasts with curcumin conjugation in rats. Compared to rats, the reduction of curcumin to hexahydrocurcumin in human intestinal and liver cytosols is 18 times and 5 times more pronounced, respectively (Xie et al., 2022).

### 2.3 Pharmacokinetics

Even when curcumin is administered intraperitoneally, its rapid drainage into the portal circulation subjects it to hepatic metabolism before it can reach systemic circulation, resulting in extremely low plasma concentrations of the parent compound (Marczylo et al., 2009; Hoehle et al., 2006). This explains the observed discrepancy between curcumin’s promising *in vitro* efficacy and its limited *in vivo* therapeutic impact.

To overcome these barriers, substantial efforts have been made to improve curcumin’s pharmacokinetic profile. Nanoformulation approaches have demonstrated particular promise in enhancing curcumin solubility, stability, and tissue bioavailability (Maleki Dizaj et al., 2022; Yallapu et al., 2015). These include the development of nanoparticles, liposomes, micelles, solid lipid nanoparticles, polymeric nanoparticles, and curcumin-loaded exosomes, all of which have shown improved pharmacological activity in preclinical models (Lv et al., 2023; Khezri et al., 2021). Notably, nanocurcumin enhances cellular uptake and brain delivery due to its small particle size and favorable surface properties, making it especially relevant for neurological applications (Lv et al., 2023; Kakkar et al., 2023; Sabouni et al., 2023).

In addition to nanosystems, the use of bioenhancers or adjuvants has proven effective. For instance, piperine, an alkaloid from black pepper, has been shown to increase curcumin bioavailability by up to 2000% by inhibiting hepatic and intestinal glucuronidation (Pawar et al., 2021; Heidari et al., 2023). Other strategies include complexation with phospholipids (as in Meriva^®^), inclusion with cyclodextrins, and co-administration with oils or emulsifying agents to improve solubilization in the gastrointestinal tract (Xu et al., 2023; Shahriari et al., 2023; Pivari et al., 2022).

Despite these advancements, many of these formulations remain in experimental or early clinical stages, and there is a pressing need for standardized, scalable, and safe delivery platforms that can maintain curcumin’s biological activity *in vivo*. Future research should focus on comparative clinical studies evaluating the bioequivalence and therapeutic efficacy of these delivery systems in humans.

## 3 Epigenetics and their modulation by curcumin

**Figure 1:**
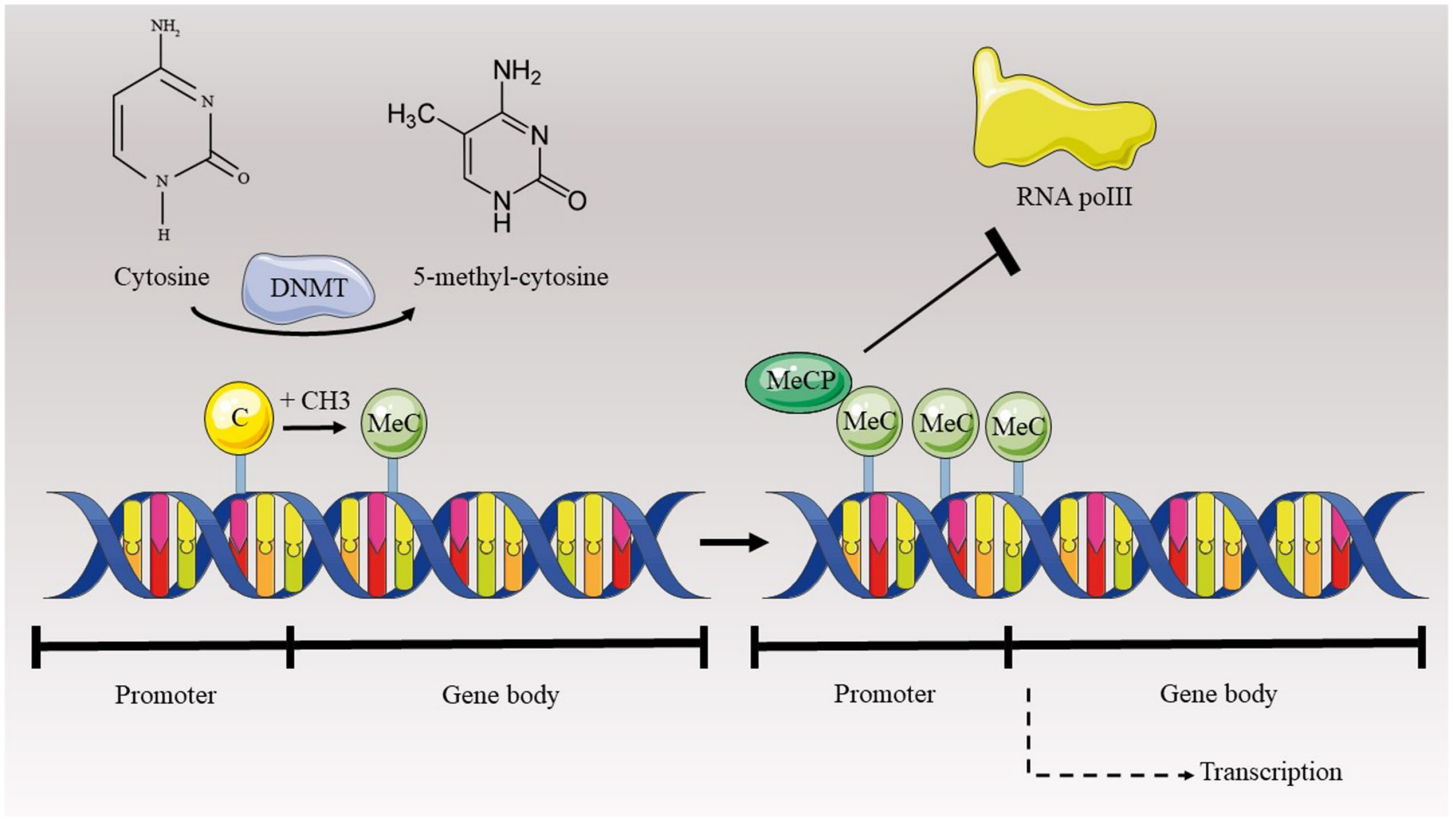
Epigenetics and biogenetics.

Epigenetics pertains to the examination of heritable and enduring alterations in gene expression that result from modifications in the chromosome structure rather than changes in the DNA sequence (Al Aboud et al., 2024). While not directly modifying the DNA sequence, epigenetic processes can change gene expression through the chemical modification of DNA bases and adjustments to the superstructure of the chromosome where DNA is enclosed. Alterations in histone proteins, which are important for organizing DNA into chromatin and regulating gene expression, can significantly impact the structure of chromatin and the accessibility of genetic material. These modifications can influence whether genes are actively transcribed or silenced, thereby affecting cellular functions such as development, differentiation, and response to environmental signals. Apart from histone modifications, DNA methylation is a form of epigenetic regulation linked to gene suppression when methylation transpires within CpG islands of promoter regions. Furthermore, non-coding RNA sequences have demonstrated a pivotal role in changing the expression of genes (Al Aboud et al., 2024). Current research indicates that epigenetics might play a crucial role in a range of health conditions, such as cardiovascular disorders, neurodegenerative diseases, and cancer. The alterations in epigenetic patterns have the potential to be altered back to their original state and might open up novel possibilities for therapy by utilizing epigenetic agents (Farsetti et al., 2023) (Figure 1). Studies showed that curcumin could modulate all of these three main epigenetic mechanisms. Therefore, we briefly discuss about these mechanisms and impact of curcumin on them.

### 3.1 DNA methylation

**Figure 2:**
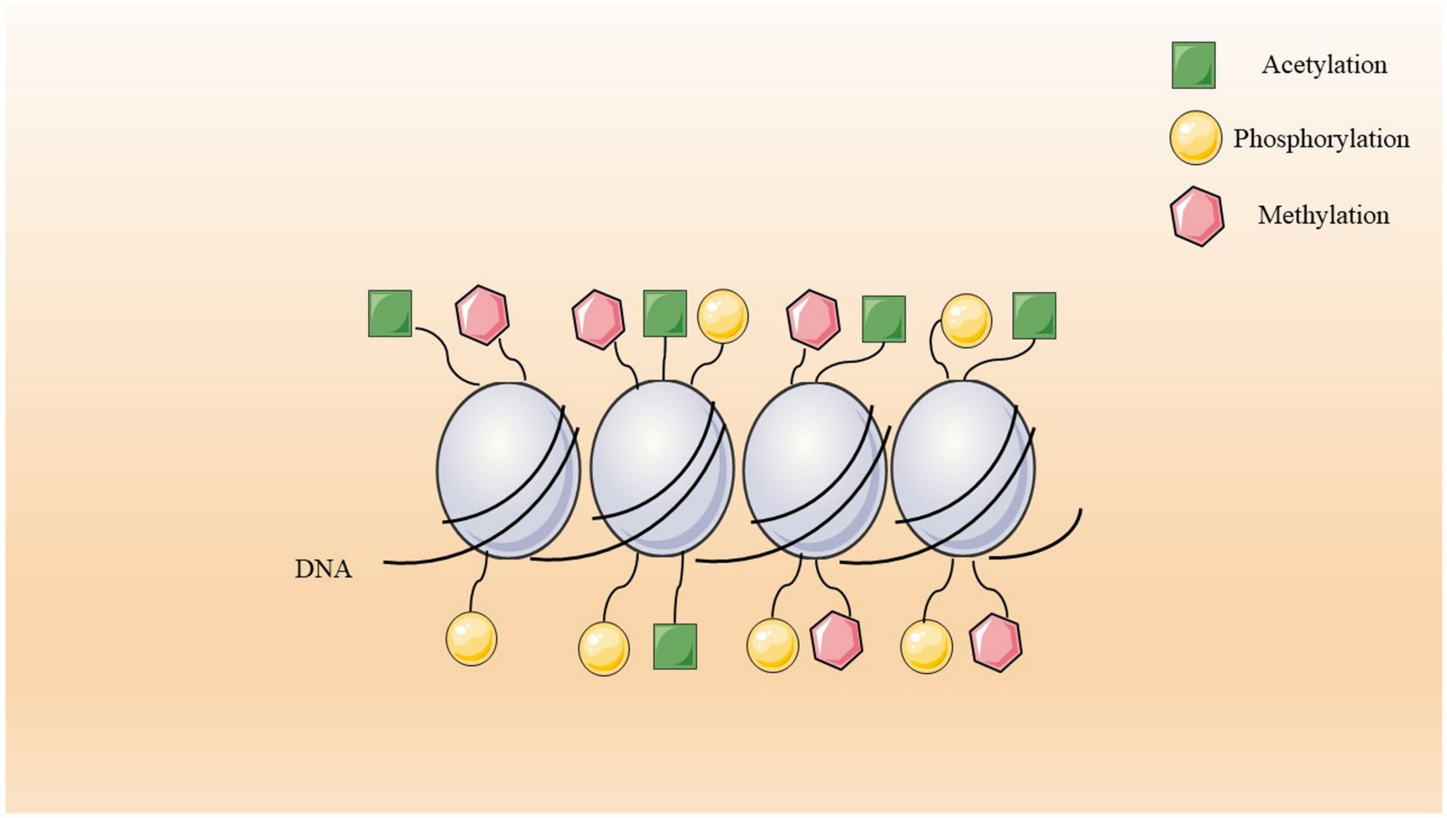
DNA methylation.

One of the heritable epigenetic modifications is DNA methylation which is characterized by the enzymatic addition of a methyl group to the C-5 position of cytosine within the DNA molecule by DNA methyltransferases (DNMTs) (Robertson, 2005). In mammals, DNA methylation is a widespread phenomenon across various genomic sequences (Lister et al., 2009). Notably, the predominant form of DNA methylation occurs within CpG dinucleotide contexts in somatic cells. However, a significant proportion of DNA methylation is observed in non-CpG contexts in embryonic stem cells (ESCs) (Lister et al., 2009). CpG dinucleotides are distributed throughout the genome, encompassing both coding and non-coding regions, with a propensity to form clusters known as CpG islands. Notably, approximately 70% of these islands are situated within the promoter regions of genes, and about half of the human genes initiate transcription from CpG sites (Deaton and Bird, 2011; Vavouri and Lehner, 2012). Methylation occurring in the promoter region is observed to impede the interaction of transcription factors with the DNA sequence beneath. The hypermethylation of promoters typically correlates with reduced gene expression or transcriptional silencing (Bird, 2002). Conversely, hypomethylation is associated with active transcription and heightened gene expression levels (Zhang et al., 2020). In contrast to promoter methylation, methylation within gene bodies and intergenic regions does not lead to transcriptomic silencing but exhibits a more nuanced relationship that varies across genes (Lorincz et al., 2004). It is proposed that methylation of CpG sites within gene bodies serves to prevent erroneous transcription factor binding and regulate alternative splicing processes (Wang Q. et al., 2022) (Figure 2).

DNA methylation profiles exhibit significant specificity to different tissues and cell types, underscoring its crucial involvement in typical ontogenic processes. The process of DNA methylation facilitates the precise activation of lineage-specific genes at particular developmental time points, while concurrently repressing pluripotency genes during the initial embryonic phases, ensuring the accurate establishment of gene expression profiles critical for the distinct development of various tissues and cell types (Gopalakrishnan et al., 2008). Moreover, DNA methylation serves as a pivotal mechanism in governing the expression of imprinted genes, enabling the allele-specific expression of gene clusters that are indispensable for normal ontogenesis (Messerschmidt et al., 2014). Indeed, the regulatory role of DNA methylation in normal developmental processes is paramount, influencing critical mechanisms such as genomic imprinting, inactivation of the X chromosome, and the regulation of repetitive element transcription and transposition. Conversely, aberrations in DNA methylation patterns have been implicated in pathological conditions, such as cancer (Gopalakrishnan et al., 2008; Jin et al., 2008; Jin et al., 2009; Bararia et al., 2023; Endo et al., 2021).

The diverse functions of DNA methylation are orchestrated by a family of DNMTs, encompassing DNMT1, DNMT2, DNMT3A, DNMT3B, and DNMT3L, each exhibiting distinct preferences and roles in the maintenance and establishment of DNA methylation patterns (Tajima et al., 2022; Qin et al., 2021; Chao et al., 2022; Adamczyk-Grochala et al., 2023; Lietz et al., 2022; Pires et al., 2023; Liu et al., 2024). While DNMT1 is primarily implicated in maintaining DNA methylation fidelity during replication, DNMT2 methylates transfer RNA molecules (Li, 2002; Li et al., 1992). The *de novo* methyltransferases DNMT3A and DNMT3B exhibit a preference for unmethylated CpG sites and are pivotal in establishing methylation patterns during developmental stages. DNMT3L acts synergistically with DNMT3A and DNMT3B to augment their enzymatic activity and facilitate binding to the methyl donor, S-adenosyl-L-methionine (SAM), although DNMT3L itself lacks catalytic function (Li, 2002; Okano et al., 1999). Recent studies suggest a level of functional interplay among DNMT1, DNMT3A, and DNMT3B in mediating both de novo and maintenance methylation processes, challenging the traditional categorization of these enzymes into distinct roles during DNA methylation dynamics (Riggs and Xiong, 2004). Liu et al. (2009) showed that curcumin covalently blocks the catalytic thiolate of DNMT1 and further inhibits DNA methylation. Furthermore, disruption of attachment of NF-κB/SP1 complex to DNMT1 promoter region is another suggested mechanism for DNA methylation regulation by curcumin (Du et al., 2012) (Table 1).

**Table 1:** Studies investigating the role of curcumin as the modulator of DNA methylation in age-related diseases.

Disease	Curcumin dosage	Cell type/ study model	Findings	Ref.
Osteoarthritis	-	Chondrocytes	Curcumin attenuates osteoarthritis by targeting miR-124/NF-kB and miR-143/ROCK1/TLR9 axis	Qiu et al. (2020)
Diabetic retinopathy	25 μM for 6 h	Insulin-deficient Ins2 Akita mice	Curcumin restores ROS production as well as DNMT functions	Maugeri et al. (2018)
Alzheimer’s disease	5 μM CUR for 48 h	N2a cells	Curcumin suppresses the AKT/NF-κB pathway via CpG demethylation of the promoter and restoration of NEP	Deng et al. (2014)
Atherosclerosis	5 μM	Vascular smooth muscle cells	Curcumin induces cell cycle arrest by SIRT7 inhibition and upregulation of DNMT2	Lewinska et al. (2015)

### 3.2 Histone modification

A crucial constituent of nucleosomes is histone proteins which undergo post-translational modifications that influence chromatin organization (Dekker and Haisma, 2009). There are six primary histone types, including H1, H2A, H2B, H3, H4, and H5, characterized by high levels of positively charged amino acids, particularly lysine and arginine. These histones exhibit remarkable evolutionary conservation, with nearly 100% sequence homology across all eukaryotic species. The nucleosome, comprising two tetrameric complexes of H2A, H2B, H3, and H4 histones forming the nucleosome core, represents the basic unit of chromatin (Onufriev and Schiessel, 2019). Linker histones H1 connect the octameric units, which are subsequently organized fibrils and further condensed into loops, helices, chromatids, or sockets. Indeed, H1 as linker histones play a critical role in chromosome stabilization, contributing to the formation of higher-order chromatin structures (Fyodorov et al., 2018). Alterations in histone modifications are linked to heterochromatin assembly and transcriptional activation processes. The structural configuration of histones influences DNA-associated functions such as transcription. Euchromatin, with a looser structure, promotes transcription of highly expressed genes, whereas heterochromatin is characterized by a denser arrangement. Epigenetic modifications, like acetylation and methylation, serve as diagnostic markers for distinct chromatin states (Neganova et al., 2022). Up to now, several different types of modifications have been identified to regulate the state of chromatin, leading to modulation of transcription and providing a suitable state of chromatin throughout cell proliferation (Neganova et al., 2022; Kundu et al., 2017; Poepsel et al., 2018).

Acetylation, methylation, phosphorylation, and ubiquitination are the most extensively researched and critical kinds of histone modifications that regulate chromatin structure and gene activity (Healy et al., 2012; Sawicka and Seiser, 2012; Rossetto et al., 2012; Swygert and Peterson, 2014; Bannister and Kouzarides, 2011). Typically, histone modifications are facilitated by specific enzymes that primarily target the histone N-terminal tails, which contain amino acids like lysine, arginine, serine, threonine, and tyrosine. However, some types of histone modifications, such as a variety of histone phosphorylation, do not follow the mentioned process. Histone acetylation typically enhances gene expression levels, although this effect may not be consistent for histone H4 (Gansen et al., 2015; Kurdistani et al., 2004; Agricola et al., 2006). Conversely, histone methylation can have either activating or repressive effects on transcription, determined by the specific amino acid positions modified in the histone tail and the number of methyl groups introduced (Swygert and Peterson, 2014; Bannister and Kouzarides, 2011; Harb et al., 2016; Potaczek et al., 2017; Morera et al., 2016; Sawicka et al., 2014). Studies have shown that dysregulation in each of the pathways and modifications may lead to a wide variety of diseases, such as cancer, atopy, and AD (Alaskhar Alhamwe et al., 2018; Santana et al., 2023). For instance, the dysregulation of histone modifications, including methylation, acetylation, sumoylation, glycosylation, phosphorylation, poly-ADP-ribosylation, and ubiquitination, can lead to the initiation and progression of cancer by modulating the activity of oncogenes or tumor suppressors (Neganova et al., 2022; Urrutia et al., 2021; Zhang et al., 2023; Montero-Hidalgo et al., 2024). Curcumin modulates histone modifications primarily by acting as a histone deacetylase (HDAC) inhibitor, meaning it prevents the removal of acetyl groups from histone proteins, leading to increased histone acetylation and ultimately influencing gene expression by making chromatin more accessible for transcription factors; this effect is considered a key mechanism in curcumin’s potential anti-cancer and anti-inflammatory properties (Hu et al., 2017). Curcumin directly inhibits the activity of several HDAC enzymes, like HDAC1, HDAC3, and HDAC8, which results in increased acetylation of histone proteins, particularly histone H3 and H4 (Hu et al., 2017).

### 3.3 Non-coding RNAs

**Figure 3:**
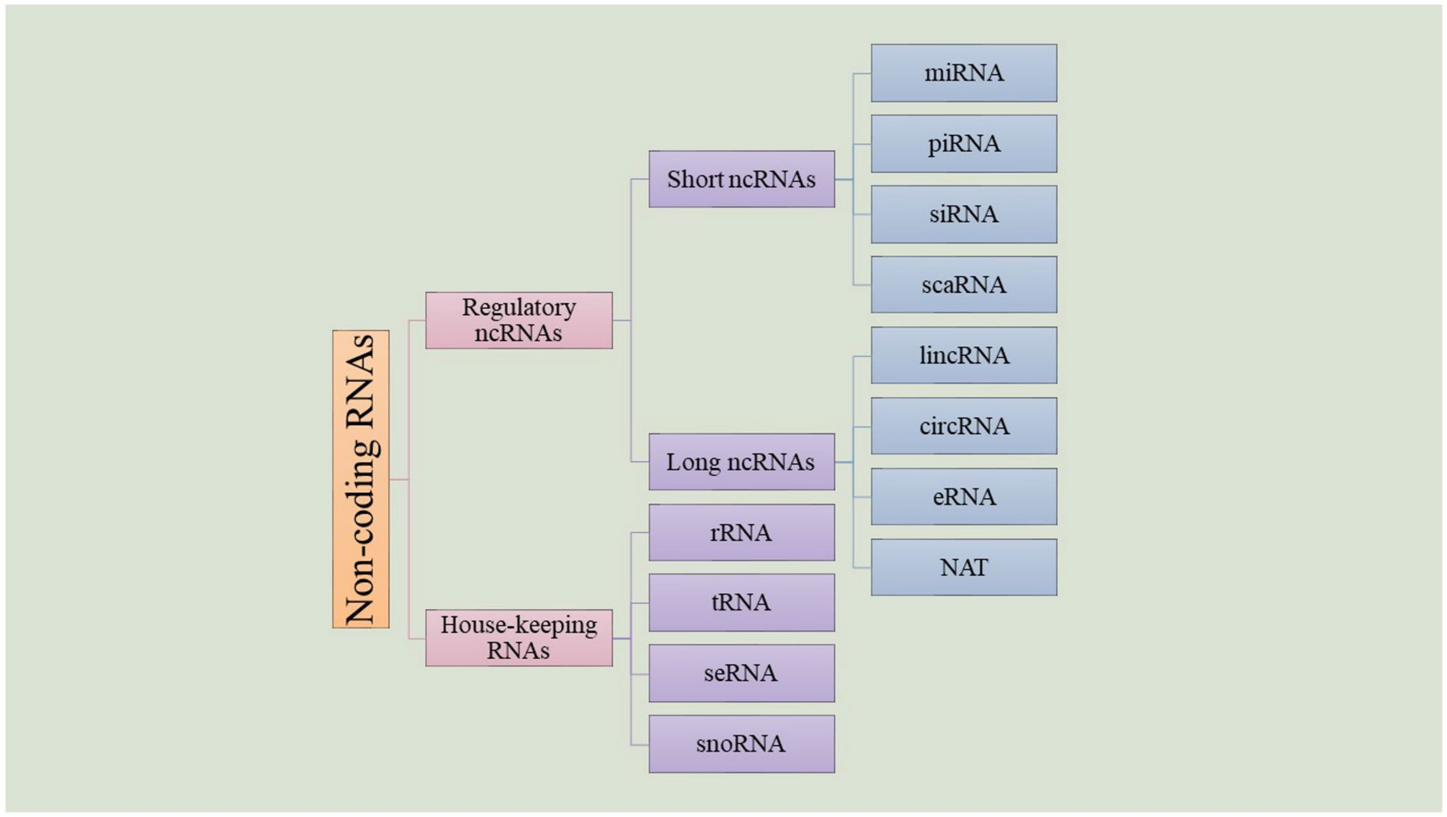
Non-coding RNAs.

Despite their name, non-coding RNAs (ncRNAs) are functional regulatory RNAs that take part in gene expression without making any alterations on the genome (Nemeth et al., 2024). Primarily, ncRNAs were discovered in 1965 by Holley and colleagues while working on a yeast (Holley et al., 1965). New research indicates that even though around 66% of the mammalian genome is involved in transcription, only about 1.9% of their genome codes for proteins and the rest are transcribed into non-coding RNAs (Nemeth et al., 2024). The exact amount of ncRNAs in the human genome is not still clear, but recent studies indicate that there are thousands of these transcripts. Many of these new ncRNAs do not have a known function and it remains a question whether most of them are just “junk RNA” or if they play a role in cellular processes which will be discovered in the years to come. This wide range of RNAs can be classified according to their function. There are two types of ncRNAs: housekeeping and regulatory RNAs. Housekeeping RNAs include ribosomal RNA, transfer RNA, small nuclear RNA, small nucleolar RNA, telomerase RNA, etc. On the other hand, regulatory ncRNAs also contain some subgroups: ncRNAs with a size of 200 nucleotides or less are considered small ncRNAs (sncRNA), and ncRNAs with more than this amount are known as long ncRNAs (lncRNAs) (Zhang P. et al., 2019). Each of these groups is categorized into subgroups that differ from each other not only by size but also by their functions and roles in cellular processes (Figure 3).

#### 3.3.1 MicroRNA

MicroRNAs (miRNAs) are the most studied type of sncRNAs which can be found in many living and non-livings (viruses) and are known to have effective parts in gene expression at the mRNA level (Lu and Rothenberg, 2018; Bhaskaran and Mohan, 2014). The development of miRNAs occurs through different steps: In the first step, RNA polymerase II (Pol II) transcribes a large primary miRNA (pri-miRNAs) from specific genes. Pri-miRNAs consist of one or a few stem-loop structures with about 70 nucleotides each which are in the nucleus (Bhaskaran and Mohan, 2014; Saliminejad et al., 2019). The next step which also occurs in the nucleus, is the cleavage of pri-miRNAs by Drosh into precursor miRNAs (pre-miRNAs). Afterward, the pre-miRNAs are translocated into the cytoplasm by means of Exportin5 (XPO5) and under the effect of an endoribonuclease named Dicer transform into small double-stranded RNAs (dsRNAs) (Bhaskaran and Mohan, 2014; Pozniak et al., 2022; Nervi and Grignani, 2014). The double-stranded microRNA then is loaded into a protein named argonaute. The combination of dsRNA and argonaute protein makes a complex that promotes the assembly of a ribonucleoprotein complex and is called RNA-induced silencing complex or RISC. After all, microRNAs are matured and target the 3′ end mRNAs and thereby, affect protein production and gene expression (Lu and Rothenberg, 2018; Saliminejad et al., 2019; Nervi and Grignani, 2014). Curcumin modulates miRNAs by directly interacting with transcription factors that regulate miRNA expression, thereby influencing the transcription of specific miRNAs, as well as by affecting epigenetic modifications like DNA methylation at miRNA promoter regions (Wan Mohd Tajuddin et al., 2019). Mechanistically, curcumin can bind to transcription factors like NF-κB, AP-1, and p53, which are known to regulate the expression of various miRNAs, leading to either upregulation or downregulation of specific miRNAs depending on the cellular context (Zhou et al., 2017). Furthermore, changing in the pattern of DNA methylation (as discussed above) by curcumin could also lead to the activation or inhibition of certain miRNAs (Wan Mohd Tajuddin et al., 2019; Mahmoudi et al., 2023).

#### 3.3.2 Small interfering RNA

siRNAs are another group of sncRNAs that mostly contain 20–25 nucleotides and have many common characteristics with miRNAs. Initially, two main differences distinguished miRNAs and siRNAs (Alshaer et al., 2021; Zhang M. M. et al., 2021). First, miRNAs were seen as naturally occurring products expressed by an organism’s genome, while siRNAs were believed to mainly come from external sources like viruses, transposons, or transgenes. Second, miRNAs were thought to be processed from stem-loop precursors with partially double-stranded features, while siRNAs were found to be derived from long, fully complementary double-stranded molecules (Zhang M. M. et al., 2021; Friedrich and Aigner, 2022). Gene silencing by siRNAs is mostly similar to the mechanisms by which miRNAs exert their effects. In the first step, long dsRNAs which have an exogenous origin, are cleaved by Dicer (Friedrich and Aigner, 2022; Carthew and Sontheimer, 2009). The next step occurs inside cells when siRNAs enter the cell and get incorporated into proteins to form the RISC (Alshaer et al., 2021; Friedrich and Aigner, 2022). After joining the RISC complex, a single-stranded siRNA is produced which searches for a matching mRNA within the RISC complex. When this siRNA links to its mRNA target, it triggers the mRNA to be cleaved, and all this leads to identifying the cut mRNA as a defect. As a result, the transcribed mRNA is degraded and no protein is translated from it (Carthew and Sontheimer, 2009; Ahn et al., 2023). Combination of curcumin and siRNAs could have synergistic effects on several preclinical cancer models (Sanati et al., 2023).

#### 3.3.3 LncRNAs

Long non-coding RNAs contain a wide range of RNAs and their classification is still somehow challenging. These RNAs can be classified according to their length, mRNA resemblance, association with repeats, biochemical pathway, etc. (St Laurent et al., 2015). Besides their vague classification, there are numerous potential ways in which lncRNAs impact chromatin changes and structure, ultimately affecting transcription or other chromatin-related processes through epigenetic means, which have been studied. Interacting with transcription factors including definitive endoderm-associated lncRNA1 (DEANR1), rhabdomyosarcoma-associated transcript (RMST), STAT3, etc. as well as chromatin remodeling are just some of their cellular effects (Lorenzi et al., 2019; Onoguchi-Mizutani and Akimitsu, 2022; Schmitz et al., 2016). Other than that, a more interesting function of these RNAs is gene silencing in post-transcriptional levels through binding to splicing factors such as PTBP1. All of the mentioned roles of lncRNAs have made them great candidates for easing the prognosis, diagnosis, and treatment, and decreasing drug resistance in many diseases (Lorenzi et al., 2019; Onoguchi-Mizutani and Akimitsu, 2022; Schmitz et al., 2016). For instance, cancer (Chu et al., 2020; Li et al., 2019), neurodegenerative diseases (Balusu et al., 2023; Plewka and Raczynska, 2022; Sekar et al., 2022), diabetes (Li et al., 2022b; Tang et al., 2023), and cardiovascular diseases (Lozano-Vidal et al., 2019; Shi and Yang, 2016; Yu et al., 2021) are only some of the diseases in which lncRNAs are studied. Curcumin could effect on the expression of different types of lncRNAs and further resulted in the inhibition or activation of a specific signaling pathway as it would be discussed in following sections (Cai et al., 2021; Liu et al., 2016).

**Figure 4:**
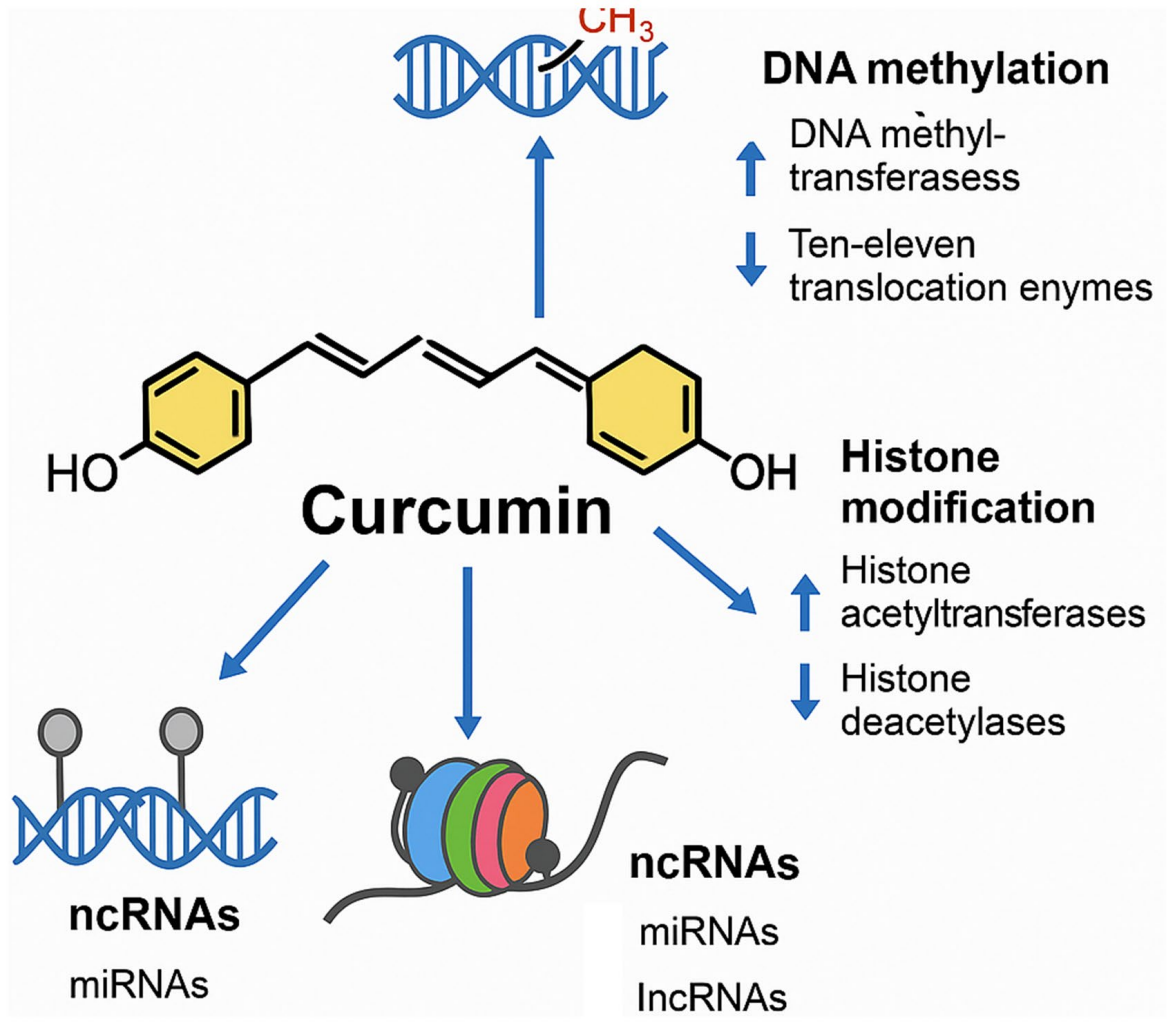
Schematic representation of curcumin’s effects on epigenetic regulation. Curcumin modulates DNA methylation by inhibiting DNA methyltransferases and enhancing TET enzyme activity, reduces histone deacetylase function to increase histone acetylation, and regulates non-coding RNAs (ncRNAs) by upregulating protective microRNAs (miRNAs) and downregulating pathogenic long non-coding RNAs (lncRNAs). These combined actions contribute to curcumin’s gene-regulatory and neuroprotective effects.

Figure 4 provides an overview of these three main epigenetic mechanisms and impact of curcumin on them.

## 4 Curcumin roles in modulating epigenetic changes in age-related diseases

### 4.1 Diabetes

Several studies have explored the epigenetic modulation by curcumin in diabetic complications, particularly its influence on DNA methylation, histone modifications, and non-coding RNAs. For instance, curcumin has been shown to reduce DNMT3A expression by 50% in insulin-deficient *Ins2 Akita* mice, suggesting a potential role in mitigating hyperglycemia-induced retinal epigenetic alterations (Maugeri et al., 2018). However, while this animal model provides insights into mechanistic pathways, its clinical translation remains uncertain, as similar reductions in DNMT activity and ROS production in human retinal pigment epithelium have not been consistently observed (Ding et al., 2022).

*In vitro* studies further suggest that curcumin inhibits NF-κB binding and decreases histone acetylase activity in monocytes exposed to high glucose (Yun et al., 2011), potentially lowering vascular inflammation. Yet, these findings are based on immortalized cell lines, which do not fully replicate the complex cellular milieu of human diabetic vasculature. Moreover, dose–response relationships and curcumin’s bioavailability were not addressed, limiting the translational value.

Similarly, Wu et al. (2022) reported that curcumin downregulated IL-17A target genes and their associated histone acetylation in diabetic retinopathy models. While promising, this study lacked controls that would clarify whether curcumin’s effects were IL-17 specific or part of broader anti-inflammatory mechanisms. The therapeutic efficacy of curcumin also remains constrained by its limited absorption and rapid metabolism *in vivo*.

C66, a curcumin analog, demonstrated reduced renal fibrosis in diabetic mice through epigenetic modifications of p300/CBP HAT expression and H3K9/14 acetylation (Wang et al., 2015). Though compelling, the use of analogs like C66 complicates direct extrapolation to native curcumin, and these findings require validation in larger animal cohorts and human studies to assess safety and pharmacodynamics (Tikoo et al., 2008).

In diabetic encephalopathy models, curcumin improved cell viability and downregulated pro-inflammatory cytokines while increasing anti-inflammatory miR-218-5p (Cui et al., 2022). While these molecular alterations are noteworthy, the *in vitro* nature of the study in PC12 cells limits its applicability to human neuronal contexts. Moreover, such single-pathway-focused findings do not account for the multifactorial nature of diabetic neurodegeneration.

Several studies also reported curcumin’s regulation of miRNAs, such as miR-152-3p in diabetic foot ulcers (Cao et al., 2024) and miR-489 in glucose-fluctuated HEK-293 cells (Fu et al., 2021). Although these studies support a regulatory role of curcumin in epigenetic networks, many rely on small sample sizes or single time-point analyses, limiting their robustness. More importantly, systemic factors influencing miRNA regulation in diabetic patients were not explored, making it unclear how these in vitro findings reflect real-world disease complexity.

Additionally, curcumin’s modulation of miR-124 in diabetic podocytes (Li et al., 2013) supports a protective role against adhesion dysfunction. However, the mechanistic specificity of curcumin’s action on miR-124 versus other competing miRNAs remains unclear. More comprehensive *in vivo* profiling is necessary to delineate the full epigenetic landscape altered by curcumin.

Taken together, while current studies highlight curcumin’s multifaceted epigenetic modulation in diabetes, most are limited by small sample sizes, short study durations, and poor clinical translation. A recurring issue is the bioavailability of curcumin, which may significantly diminish its therapeutic efficacy in humans compared to experimental models. Future research should prioritize clinical trials, pharmacokinetic assessments, and comparative studies with standard treatments to clarify curcumin’s true therapeutic potential in diabetes-related epigenetic regulation.

### 4.2 Cardiovascular diseases and hypertension

Curcumin has been extensively investigated for its role in cardiovascular diseases (CVD) through epigenetic and inflammatory pathways. However, despite promising preclinical data, several studies lack rigorous validation in clinical models and suffer from limited translational impact.

In vascular smooth muscle cells (VSMCs), curcumin demonstrated cytostatic effects and upregulated p21 and p53, potentially through SIRT7 suppression and DNMT2 upregulation (Lewinska et al., 2015). These findings suggest transcriptional silencing of rDNA and stabilization of RNA. Nevertheless, the study’s reliance on *in vitro* VSMC cultures, without parallel *in vivo* models, restricts its generalizability. Furthermore, the DNA damage-independent p53 induction observed contradicts typical stress-induced apoptotic pathways, suggesting a need for further mechanistic clarification.

Morimoto et al. reported that curcumin disrupts p300/GATA4 complexes and inhibits hypertrophy in rat cardiomyocytes (Morimoto et al., 2008). Although in vivo experiments supported reductions in myocardial thickness, functional outcomes like exercise tolerance or survival were not assessed. Additionally, these rodent models may not fully recapitulate the heterogeneity of human heart failure, especially in aged or comorbid populations.

In the context of atherosclerosis, the TFEB-p300-BRD4 axis was shown to mediate the inflammatory response in macrophages exposed to ox-LDL (Remmerie and Scott, 2018; Li et al., 2022a). Curcumin restored autophagy and lipid metabolism via TFEB nuclear translocation. However, a critical limitation of this model is the absence of in vivo validation of TFEB’s role as a mediator in curcumin’s activity. Moreover, while super-enhancer modulation by curcumin appears promising, this remains a relatively unexplored area requiring deeper molecular mapping and longitudinal outcome studies.

Nanocurcumin has shown benefits in hypertrophied cardiomyocytes by modulating mitochondrial stress markers and preventing substrate switching (Nehra et al., 2015). Despite advanced bioengineering approaches, the lack of comparative data with standard therapies (e.g., ACE inhibitors) raises questions about therapeutic relevance. Moreover, the use of nanocarriers, though advantageous for bioavailability, introduces complexity regarding toxicity, clearance, and regulatory approval.

In spontaneous hypertensive rats (SHRs), curcumin reduced TGFβ, HDAC1, and MMP-2, promoting histone acetylation at the TIMP1 promoter (Hu et al., 2017). While these epigenetic shifts align with decreased fibrosis, the study failed to show changes in blood pressure-lowering efficacy, a primary endpoint in hypertension treatment. Similarly, Sunagawa et al. observed improvements in left ventricular (LV) mass and wall thickness in salt-sensitive rats (Sunagawa et al., 2021), but the dissociation between structural changes and systolic function raises concerns about functional benefit versus biomarker modulation.

The involvement of NLRP3 inflammasome in VSMC proliferation and vascular remodeling has been supported by both in vitro and in vivo evidence (Zhang J. et al., 2019). Curcumin was shown to inhibit NLRP3 activation via suppression of NF-κB and histone acetyltransferase activity. However, curcumin’s dose-dependent effects on inflammasome components were not adequately characterized, and the long-term cardiovascular outcomes of NLRP3 inhibition remain unexplored.

In peripheral arterial disease (PAD), curcumin increased angiogenesis by upregulating miR-93 (Geng et al., 2016). While endothelial function improvements were notable, suppression of miR-93 abolished curcumin’s benefits, indicating a narrow mechanistic focus that may limit therapeutic robustness. Similarly, in myocardial infarction models, curcumin increased miR-7a/b and decreased SP1, reducing apoptosis (Jiang et al., 2024). However, these miRNA-mediated effects are highly context-dependent and may not generalize across diverse cardiovascular injuries.

In a model of acute pulmonary embolism, curcumin alleviated myocardial injury and inflammation by upregulating miR-145-5p and targeting IRS1 (Ouyang et al., 2022). Yet, potential off-target effects of miRNA regulation were not addressed, and the therapeutic window remains unclear. Moreover, translating these findings to human pulmonary embolism is challenging due to the absence of coagulopathy, hemodynamic variables, and standard interventions in the model.

Curcumin also modulates atherosclerotic inflammation through the MIAT/miR-124 axis, as evidenced by studies in ox-LDL-treated macrophages (Chen C. et al., 2022). While this axis appears promising, the use of single-cell types without immune cell interaction or hemodynamic stress limits the system’s fidelity. Furthermore, the regulation of lncRNA/miRNA networks by curcumin requires broader transcriptomic validation.

Another mechanism involves curcumin’s modulation of exosomal miR-92b-3p, targeting KLF4 and RUNX2 to mitigate vascular calcification (Tan et al., 2021). While curcumin improved VSMC calcification markers in vitro and in animal models, human studies assessing vascular compliance or calcium scores are lacking. The therapeutic modulation of exosomal miRNAs also introduces challenges regarding dosing consistency and systemic off-target effects.

Tan et al. demonstrated curcumin-induced cholesterol efflux in THP-1 macrophages via the miR-125a-5p/SIRT6/ABCA1 axis (Xu et al., 2019). However, curcumin’s effect on lipid panels or atherosclerotic burden in vivo was not reported, limiting clinical relevance. Moreover, the study did not compare curcumin’s efficacy with statins or PCSK9 inhibitors, which are standard in lipid modulation.

In models of cerebral ischemia, curcumin influenced several miRNAs, including miR-7-5p, miR-1287-5p, and LONP2, leading to improved oxidative resilience and reduced infarct size (Zhang T. et al., 2021; Zhou et al., 2021). While compelling, these studies relied on rodent stroke models, which often fail to reproduce human heterogeneity in infarct evolution, comorbid conditions, and rehabilitation response. Moreover, miRNA-target dynamics in the ischemic brain are temporally complex, and curcumin’s acute versus long-term effects were not delineated.

Overall, while curcumin demonstrates robust epigenetic and anti-inflammatory actions in preclinical CVD and hypertension models, clinical translation remains limited due to the lack of long-term outcome studies, variability in dosage and bioavailability, and inadequate comparison with standard-of-care treatments. Many studies are conducted in small sample sizes, single animal models, or isolated cell systems, making it difficult to predict efficacy in complex human diseases. Future research must address these limitations through rigorously designed clinical trials, multi-omic analyses, and head-to-head comparisons with existing therapies to validate curcumin’s therapeutic relevance in cardiovascular health.

### 4.3 Neurodegenerative diseases and dementia

Neurodegenerative diseases such as Alzheimer’s disease (AD) and Parkinson’s disease (PD) involve complex epigenetic dysregulation. Curcumin has been shown to modulate these pathways in preclinical studies, but significant limitations hinder its translation to clinical use.

#### 4.3.1 Curcumin and its role in epigenetic modifications in AD

Alzheimer’s disease (AD) is a progressive neurodegenerative condition marked by memory loss, synaptic dysfunction, neuroinflammation, mitochondrial deficits, and accumulation of amyloid-beta (Aβ) plaques and hyperphosphorylated tau tangles (Rafiyan and Mojtahedi, 2025). Epigenetic alterations play central roles in AD pathogenesis, making them attractive therapeutic targets. Curcumin, a natural polyphenol with multi-targeted properties, has garnered attention for its capacity to modulate these pathways, offering potential neuroprotective benefits.

Curcumin influences several epigenetic mechanisms relevant to AD. It downregulates DNA methyltransferases (DNMTs), reversing the aberrant hypermethylation of neuroprotective genes such as *BDNF* and *CREB*, and mitigating the hypomethylation of pro-inflammatory genes. This contributes to restored neuronal plasticity and reduced inflammation (Prasanth et al., 2024; Abdul-Rahman et al., 2024). Curcumin also inhibits histone deacetylases (HDACs), leading to increased acetylation at key promoters that regulate learning and memory-related genes. Additionally, it modulates histone methylation by promoting demethylation of H3K27, enhancing transcription of genes involved in synaptic function and neuronal survival (Li J. et al., 2021; Wang Y. et al., 2022).

Non-coding RNAs represent another layer of epigenetic regulation affected by curcumin. It upregulates neuroprotective microRNAs (e.g., miR-132) and suppresses neuroinflammatory ones like miR-155, contributing to decreased cytokine production and improved synaptic integrity. Curcumin also influences long non-coding RNAs such as BACE1-AS and MIAT, which are implicated in Aβ accumulation and neuronal apoptosis (Wang et al., 2025; Zhao et al., 2023).

Moreover, curcumin enhances neurogenesis and synaptic plasticity, largely through the activation of the BDNF–TrkB signaling cascade and phosphorylation of CREB, a transcription factor essential for memory consolidation (Huang et al., 2015; Xu et al., 2019). It also activates peroxisome proliferator-activated receptor gamma coactivator 1-alpha (PGC-1*α*), which stimulates mitochondrial biogenesis and improves cellular energy metabolism (Reddy et al., 2016). This leads to increased ATP production and a reduction in oxidative stress via upregulation of antioxidant enzymes like superoxide dismutase (SOD) and catalase (He et al., 2025).

Curcumin’s effects extend to the pathological hallmarks of AD. It inhibits glycogen synthase kinase-3β (GSK-3β), thereby reducing tau phosphorylation. Simultaneously, it facilitates autophagic clearance of Aβ plaques by enhancing lysosomal activity and promoting microglial phagocytosis (Yang et al., 2025; Huang et al., 2014). Animal studies consistently demonstrate curcumin’s cognitive benefits, including improved spatial memory and learning, reduced plaque burden, and normalization of synaptic protein expression.

Additionally, curcumin modulates major inflammatory pathways in AD. It suppresses nuclear factor-kappa B (NF-κB) activation, leading to decreased expression of pro-inflammatory cytokines such as IL-1β, TNF-*α*, and IL-6. It also induces a shift in microglial phenotype from pro-inflammatory (M1) to anti-inflammatory (M2), further supporting neural repair and anti-apoptotic processes (Azzini et al., 2024).

Despite these promising preclinical findings, translation to human therapy faces significant barriers. The majority of existing studies are based on *in vitro* models or transgenic mice, which often fail to capture the multifactorial nature of human AD, particularly in aged populations (Rafiyan et al., 2023). Furthermore, curcumin suffers from poor bioavailability, rapid metabolism, and limited blood–brain barrier penetration—issues that limit its efficacy in clinical settings. Clinical trials to date have reported mixed outcomes, often attributed to formulation variability, short study durations, and small sample sizes (Dong et al., 2012; Ruan et al., 2022; Huang et al., 2012).

To address these limitations, recent efforts have focused on nanoformulations and liposomal carriers to improve curcumin’s pharmacokinetics and brain delivery. However, these approaches remain in early stages and require rigorous clinical evaluation. Moreover, while curcumin’s multi-targeted actions are theoretically advantageous, they may also pose risks of off-target effects or unanticipated gene modulation.

In summary, curcumin exerts significant modulatory effects on epigenetic pathways involved in AD, including DNA methylation, histone modification, non-coding RNA regulation, neurogenesis, mitochondrial function, and protein aggregation. These mechanisms contribute to improved neuronal survival, reduced inflammation, and enhanced cognitive performance in preclinical models. However, its clinical utility remains constrained by pharmacological and translational challenges. Future research should prioritize high-quality clinical trials, multi-omic validation, and optimized delivery systems to fully evaluate curcumin’s therapeutic potential in Alzheimer’s disease.

#### 4.3.2 Curcumin and epigenetic modifications in Parkinson’s disease (PD)

Parkinson’s disease (PD) is a progressive neurodegenerative disorder characterized by the selective degeneration of dopaminergic neurons in the substantia nigra, leading to motor symptoms such as bradykinesia, tremor, and rigidity, along with cognitive and mood impairments. Epigenetic dysregulation plays a significant role in PD pathogenesis, making these mechanisms appealing therapeutic targets. Curcumin, owing to its broad bioactivity, has been studied extensively for its potential neuroprotective effects in PD models.

Curcumin has been shown to influence epigenetic modifications implicated in PD. It inhibits DNA methyltransferases (DNMTs), leading to demethylation and reactivation of silenced neuroprotective genes such as *Nurr1*, *DJ-1*, and PARK genes. These genes are essential for dopaminergic neuron maintenance and mitochondrial function (Chiu et al., 2020; Giordano et al., 2014). In parallel, curcumin acts as a histone deacetylase (HDAC) inhibitor, enhancing histone acetylation and promoting the transcription of genes involved in neuronal survival and synaptic plasticity (He et al., 2025). It also reduces repressive histone methylation marks such as H3K27me3 and H3K9me2, creating a chromatin environment more favorable to neuroprotective gene expression (Prasanth et al., 2024).

Beyond chromatin remodeling, curcumin modulates non-coding RNAs, including miRNAs that regulate oxidative stress, inflammation, and proteostasis. While specific targets in PD are still emerging, early findings suggest that curcumin may restore miRNA balance disrupted in neurodegeneration, further supporting neuronal homeostasis (Cai et al., 2025).

Curcumin also exerts biogenetic effects critical to PD pathology. It enhances mitochondrial biogenesis and function by activating antioxidant enzymes such as SOD2 and catalase, thereby mitigating oxidative damage—a central feature in PD progression (Dehghani et al., 2020). Curcumin modulates the AMPK-mTOR signaling axis to enhance autophagy, facilitating the degradation of misfolded *α*-synuclein aggregates, a major pathological hallmark of PD (Khayatan et al., 2025). It further promotes lysosomal enzyme activity, including cathepsin D, improving proteostasis and reducing neurotoxicity (Jiang et al., 2013).

In preclinical PD models, curcumin improves dopaminergic function by increasing the expression of tyrosine hydroxylase, the rate-limiting enzyme in dopamine synthesis, and restoring dopamine levels in the striatum (Sang et al., 2018). It also enhances dopaminergic signaling through upregulation of D2 receptors and protects neurons via activation of PI3K/Akt and ERK pathways, which are involved in cell survival and synaptic integrity (Naser et al., 2022).

Behaviorally, curcumin has demonstrated the ability to improve motor deficits and cognitive impairment in animal models of PD. It reduces neuroinflammation by inhibiting NF-κB and pro-inflammatory cytokines such as TNF-α, IL-1β, and IL-6. Moreover, it activates the Nrf2-ARE pathway, enhancing antioxidant responses and reducing oxidative stress (Nebrisi, 2021; Cui et al., 2022; Sharma and Nehru, 2018). Curcumin also inhibits α-synuclein aggregation by directly interacting with fibrils and promoting their disassembly, contributing to preserved neuronal function (Sharma and Nehru, 2018).

Despite these promising results, the translation of curcumin’s effects to clinical use remains challenging. Most evidence arises from in vitro studies or toxin-induced rodent models that do not fully replicate the complex, chronic, and age-related nature of human PD. Moreover, curcumin’s poor bioavailability and limited blood–brain barrier penetration significantly restrict its therapeutic efficacy. While nanoformulations such as Lipocurc™ have shown improved delivery and motor benefits in PD models (Chiu et al., 2013), these approaches are still in the early stages of clinical investigation.

There is also a lack of large-scale human trials assessing curcumin’s impact on PD progression or symptom management. The available data often lack standardization in dosing, delivery systems, and outcome measures, making it difficult to draw firm conclusions. Furthermore, curcumin’s broad range of molecular targets, while beneficial in theory, may lead to unpredictable effects or off-target consequences in complex human neurobiology (Liu et al., 2019; Gong and Sun, 2022).

In conclusion, curcumin exhibits neuroprotective effects in PD models through a combination of epigenetic regulation, mitochondrial support, anti-inflammatory action, and enhancement of autophagy and dopaminergic signaling. These effects contribute to improved neuronal survival, reduced α-synuclein pathology, and better motor and cognitive function in experimental settings. However, curcumin’s clinical translation is limited by pharmacokinetic constraints, model-system gaps, and a lack of robust human data. Future research should focus on optimizing bioavailability, clarifying target specificity, and conducting well-designed clinical trials to assess its therapeutic potential in Parkinson’s disease.

### 4.4 Other diseases

#### 4.4.1 Osteoarthritis

In osteoarthritis cells, exosomes that are derived from curcumin-treated MSCs suppress apoptosis and maintain cell viability. These exosomes also restore the expression of miR-124 and miR-143 which are downregulated in osteoarthritis. Furthermore, the exosomes derived from curcumin-treated MSCs modulate the expression of ROCK1 and NF-kB which are increased in osteoarthritis. Further investigations have indicated that curcumin administration leads to DNA methylation at promoter regions of miR-124 and miR-143. Besides, 3′UTRs of ROCK1 and NF-kB have shown sites to bind to miR-124 and miR-143, respectively. Altogether, curcumin leads to the production of exosomes that upregulate miR-124 and miR-143, preventing the progression of osteoarthritis (Qiu et al., 2020).

#### 4.4.2 Arthritis

A study conducted on synovial fibroblasts has shown that in the promoter region of IL-6, histone modification levels are increased in patients with rheumatoid arthritis compared to patients with osteoarthritis. Thus, it is suggested that in rheumatoid arthritis synovial fluid, the structure of chromatin is in a loose or open state. On the other hand, treating cells with curcumin is found to significantly decrease the H3ac level in the promoter region of IL-6 while reducing IL-6 expression at mRNA and protein levels (Wada et al., 2014). In rheumatoid arthritis, fibroblast-like synovial (RAFLS) cells, curcumin increases apoptosis while suppressing growth, invasion, and migration. Furthermore, it inhibits cells’ inflammatory responses. Linc00052 levels are increased following curcumin treatment which serves as a regulator of the protein inhibitor of activated STAT 2 (PIAS2) through sponging miR-126-5p. In addition, curcumin suppresses the signal transducer and activator of transcription 3 (STAT3) and Janus kinase 2 (JAK2) pathways. *In vivo* studies also revealed that curcumin alleviates inflammatory infiltration, proliferation of synovial cells, and arthritis score (Xiao et al., 2022).

#### 4.4.3 Osteoporosis

In dexamethasone-induced osteoporosis, hypercalciuria is observed in mice; whereas, treating them with curcumin leads to a reduction in calcium in urine. Curcumin administration is able to abolish dexamethasone-mediated resorption of bone, as evidenced by a decrease in CTX and TRAP-5b which are bone resorption markers as well as an increase in serum levels of OCN. Further investigations revealed an increased separation in the trabecular bone network. In addition, trabecular thickness in the tibia’s proximal metaphysis has been reduced in mice with osteoporosis. However, curcumin is indicated to reverse the mentioned adverse effects and induce bone remodeling. Indeed, curcumin is found to reverse miR-365 downregulation in the tibia which regulates MMP9. Therefore, it is suggested that curcumin exerts protective roles in osteoporosis partially by targeting miR-365 which consequently leads to the suppression of MMP9 (Li et al., 2015).

#### 4.4.4 COPD

Histone deacetylase-2 (HDAC2) is found to impair the lungs of patients suffering from COPD. Furthermore, corticosteroids exert their anti-inflammatory roles partly through HDAC2. Meja and colleagues have indicated that at nanomolar concentrations, curcumin is able to restore the activity of HDAC2 which is impaired by the extract of cigarette smoke or oxidative stress. Furthermore, it can restore the corticosteroids’ anti-inflammatory roles at a concentration of 200 nM. Interestingly, this restoring function of curcumin on the expression of HDAC2 works even when a protein synthesis inhibitor, such as cycloheximide, is present. Further studies have also revealed that up to muM of curcumin exerts its roles through a pathway that is not dependent on anti-oxidative and rather related to the phosphorylation-ubiquitin-proteasome axis (Meja et al., 2008). Findings of an investigation have revealed that in the type II alveolar epithelial cells (AEC II) of the rat COPD model, mRNA levels of MIP-2α, MCP-1, and IL-8 are upregulated compared to the control group. Meanwhile, HDAC2 protein expression has been significantly lower compared to the control group and has been negatively correlated to the increased levels of MIP-2α, MCP-1, and IL-8. Furthermore, cells of the COPD model showed higher acetylation levels of H3/H4 and lower methylation levels of H3K9 in the promoter region of different chemokine genes. Curcumin is able to abolish the mentioned changes while restoring the expression of HDAC2, reducing the acetylation levels of H3/H4, and increasing the methylation of H3K9 (Gan et al., 2016).

#### 4.4.5 Nephrosclerosis

A study regarding the effects of curcumin on nephrosclerosis has found that rates with a high-salt diet whether they received curcumin or not show an increase in their systolic blood pressure. However, serum creatinine level is only increased in rats with high-salt diet rats. Curcumin treatment is found to suppress the fibrosis and inflammation which occur in nephrosclerosis. Furthermore, it suppresses histone acetylation at Lys 9 which is increased in rats with a high-salt diet. In addition, the chromatin immunoprecipitation test demonstrated that the expression of IL-6 is correlated with acetylated H3K9. Therefore, it is implied that curcumin improves nephrosclerosis by suppressing the acetylation of histones (Muta et al., 2016).

#### 4.4.6 Cataracts

In SRA01/04 cells, curcumin decreases transforming growth factor-β2 (TGF-β2)-mediated invasion, migration, and proliferation. In patients with posterior capsule opacification, KCNQ10T1 is increased. Whereas, curcumin’s beneficial effects in TGF-β2-induced SRA01/04 cells are abolished by overexpression of KCNQ10T1. Indeed, KCNQ10T1 is negatively correlated to the miR-377-3p which interacts with COL1A2 3′ untranslated region (3′UTR). Therefore, it is concluded that curcumin plays a protective role against adverse changes in lens epithelial cells through KCNQ1OT1/miR-377-3p/COL1A2 signaling (Huai et al., 2022).

## 5 Current imitations and future perspectives

Although a number of studies demonstrate that curcumin has beneficial effects on epigenetic modulation and therapeutic potential for age-related diseases, several significant limitations impede its clinical translation. These challenges are primarily associated with curcumin’s poor bioavailability, inconsistent results in clinical studies, the lack of standardized doses, and limited understanding of its mechanism of action in human models (Anand et al., 2007). Addressing these issues is essential for curcumin’s successful incorporation into clinical therapies.

### 5.1 Pharmacokinetic limitations and poor bioavailability

One of the most significant limitations of curcumin is its poor bioavailability. Curcumin has low water solubility (with about 11 ng/mL in alkaline conditions), undergoes extensive metabolism in the liver and intestines, and is rapidly eliminated from the body, forming various metabolites (e.g., glucuronides, sulfates) that result in low plasma concentrations (Anand et al., 2007; Hussain et al., 2022; El Oirdi and Farhan, 2024; Lopresti, 2018). Additionally, curcumin has a short half-life, which further limits its therapeutic potential (Henrotin et al., 2010).

To overcome these limitations, several strategies have been developed.

#### 5.1.1 Nanoparticle drug delivery systems

Nanocurcumin, polymeric nanoparticles, and liposomal curcumin enhance solubility and increase systemic absorption (Szymusiak et al., 2016; Shojaei et al., 2023). Szymusiak et al. (2016) developed nanocurcumin for using in CNS of mice and found significant increase of bioavailability compared to curcumin suggested the potential therapeutic application of this method. Also, curcumin is a lipophilic agent and load liposomes with curcumin could enhance its bioavailability significantly (Hegde et al., 2023; Tønnesen et al., 2002). In another study, Chen W. T. et al. (2022) showed significant improvement of bioavailability of curcumin and its antibacterial effects by curcumin-loaded liposomes.

#### 5.1.2 Curcumin analogs

Chemically modified curcumin derivatives like EF24 and GO-Y030 show improved stability and bioactivity (Sato et al., 2011; Wu et al., 2017; Adams et al., 2005). Adams et al. (2004) developed EF24 as an analog of curcumin and showed its high potential against inflammation and cancer. Further studies increased the bioavailability of these analogs; as an example, Wu et al., developed another analog of EF24 with greater anti-cancer activity as well as better bioavailability than curcumin and EF24 (Wu et al., 2017; Adams et al., 2004). Shimizu et al. (2020) also developed GO-Y030 as another curcumin analog and found greater bioavailability of this analog than curcumin.

#### 5.1.3 Bioenhancers

Piperine, an extract from black pepper, has been shown to increase curcumin absorption significantly with up to about 20 times (Patil et al., 2016; Shoba et al., 1998).

### 5.2 No standardized dosage and formulation used in clinical studies

The variability in dosage, formulation, and treatment duration across clinical studies on curcumin has contributed to inconsistent results. Most preclinical studies use doses of curcumin that are many orders of magnitude higher than those achievable through food alone, complicating direct translation to human therapy (Liu et al., 2022). Moreover, various formulations, such as pure curcumin, curcumin-phytosome, and nanocurcumin, have been used in separate studies, making it difficult to compare results (Stohs et al., 2020; Gera et al., 2017). Development of standardized dosage forms that maintain stable plasma concentrations, enabling their use in clinical settings as well as refining curcumin formulations to ensure consistent and effective bioavailability across different patient populations are suggested to address these issues.

### 5.3 Translational facets and limited clinical evidence

While curcumin has shown efficacy in animal models and *in vitro* experiments, there is still a lack of large, well-controlled clinical trials to confirm its effectiveness in human populations. Most studies are limited by small sample sizes, short treatment periods, and reliance on surrogate markers rather than long-term clinical outcomes (Gupta et al., 2013; Kuszewski et al., 2018). Additionally, genetic polymorphisms affecting curcumin metabolism and disposition vary significantly across populations, which could further complicate the effectiveness of curcumin in diverse groups (Yan et al., 2018; Mukwevho and Matumba, 2019; Hassaninasab et al., 2011). These trials should evaluate the lasting effects of curcumin on age-related diseases and consider long-term clinical outcomes. Furthermore, personalized approaches could enhance curcumin’s efficacy, tailoring treatments to the individual’s genetic and metabolic profile.

### 5.4 Potential risks and concerns about long-term safety

Curcumin is generally regarded as safe, but excessive dosages or long-term use may lead to gastrointestinal discomfort and potential drug interactions with medications such as anticoagulants. Despite the fact that curcumin could be used as a reliever of gastrointestinal symptoms such as bloating and indigestion, use of high dose of curcumin could induce abdominal pain (Carroll et al., 2011). In a study conducted by Carroll et al. (2011), patients who used 8 g/d of curcumin for 2 weeks have complaints about abdominal pain and bulky volume of the tablets. Curcumin could inhibit platelet aggregation and further inhibits blood coagulation suggesting that taking it with other anticoagulants should be with cautious (Kim et al., 2012; Keihanian et al., 2018). However, some studies showed no significant interaction between antocoagulants and curcumin which suggested further studies for better evaluation of these effects (Hu et al., 2018). Some studies have also suggested that curcumin might affect iron metabolism, which could pose problems for individuals with iron-deficiency disorders (Smith and Ashar, 2019). Curcumin downregulates hepcidin production, a protein involved in iron metabolism, and in high doses or in the previous subclinical iron-deficiency anemia, it could induce iron-deficiency disorders (Jiao et al., 2009). Some case reports also suggested the potential of hepatotoxicity of curcumin and increase of liver enzymes (Halegoua-DeMarzio et al., 2023). Comprehensive data on the long-term effects of curcumin supplementation, especially in aging populations, is crucial. Additionally, understanding how curcumin interacts with commonly prescribed medications to avoid adverse effects.

## 6 Conclusion

Curcumin emerges as a promising neuroprotective agent capable of modulating epigenetic pathways to enhance cognitive function and neuroplasticity in aging and neurodegenerative diseases. Its ability to regulate DNA methylation, histone modifications, and non-coding RNAs, alongside its influence on neurogenesis, mitochondrial function, and synaptic remodeling, underscores its therapeutic potential in conditions such as AD and PD disease. Furthermore, curcumin’s anti-inflammatory and antioxidant properties contribute to its capacity to counteract neurodegeneration and promote brain resilience. Despite these compelling benefits, several challenges remain, particularly regarding curcumin’s bioavailability, effective dosing strategies, and long-term safety in human populations. Innovative drug delivery systems, including nanoparticle formulations and lipid-based carriers, are promising approaches to enhance curcumin’s stability and absorption. Additionally, combining curcumin with lifestyle interventions such as exercise and dietary modifications may further potentiate its neuroprotective effects through complementary mechanisms. Future research should focus on well-designed clinical trials to establish optimal curcumin formulations, precise molecular targets, and its long-term impact on cognitive function. Moreover, studies exploring personalized approaches that integrate curcumin with other therapeutic strategies may pave the way for more effective neuroprotective interventions. By harnessing curcumin’s epigenetic potential, we can advance novel strategies to promote cognitive longevity and mitigate the burden of neurodegenerative diseases. While this review does not present new experimental data, its novelty lies in the synthesis of current findings into a unified framework that bridges nutraceutical interventions—specifically curcumin—with epigenetic regulation in age-related diseases. By categorizing curcumin’s molecular actions into DNA methylation, histone modification, and non-coding RNA modulation, this review offers a mechanistic scaffold that connects diverse studies under a coherent epigenetic lens. This integrated perspective may help guide future experimental designs and therapeutic strategies targeting the epigenome through bioactive dietary compounds.

## References

[ref1] Abdul-Rahman T. Awuah W. A. Mikhailova T. Kalmanovich J. Mehta A. Ng J. C.. (2024). Antioxidant, anti-inflammatory and epigenetic potential of curcumin in Alzheimer's disease. Biofactors 50, 693–708. doi: 10.1002/biof.2039, PMID: 38226733

[ref2] Adamczyk-Grochala J. Bloniarz D. Zielinska K. Lewinska A. Wnuk M. (2023). *DNMT2/TRDMT1* gene knockout compromises doxorubicin-induced unfolded protein response and sensitizes cancer cells to ER stress-induced apoptosis. Apoptosis 28, 166–185. doi: 10.1007/s10495-022-01779-0, PMID: 36273376 PMC9950192

[ref3] Adams B. K. Cai J. Armstrong J. Herold M. Lu Y. J. Sun A.. (2005). EF24, a novel synthetic curcumin analog, induces apoptosis in cancer cells via a redox-dependent mechanism. Anti-Cancer Drugs 16, 263–275. doi: 10.1097/00001813-200503000-00005, PMID: 15711178

[ref4] Adams B. K. Ferstl E. M. Davis M. C. Herold M. Kurtkaya S. Camalier R. F.. (2004). Synthesis and biological evaluation of novel curcumin analogs as anti-cancer and anti-angiogenesis agents. Bioorg. Med. Chem. 12, 3871–3883. doi: 10.1016/j.bmc.2004.05.006, PMID: 15210154

[ref5] Adibian M. Hodaei H. Nikpayam O. Sohrab G. Hekmatdoost A. Hedayati M. (2019). The effects of curcumin supplementation on high-sensitivity C-reactive protein, serum adiponectin, and lipid profile in patients with type 2 diabetes: a randomized, double-blind, placebo-controlled trial. Phytotherapy Res. 33, 1374–1383. doi: 10.1002/ptr.6328, PMID: 30864188

[ref6] Agarwal R. Goel S. K. Behari J. R. (2010). Detoxification and antioxidant effects of curcumin in rats experimentally exposed to mercury. J. Appl. Toxicol. 30, 457–468. doi: 10.1002/jat.1517, PMID: 20229497

[ref7] Aggarwal B. B. Kumar A. Bharti A. C. (2003). Anticancer potential of curcumin: preclinical and clinical studies. Anticancer Res. 23, 363–398, PMID: 12680238

[ref8] Agricola E. Verdone L. Di Mauro E. Caserta M. (2006). H4 acetylation does not replace H3 acetylation in chromatin remodelling and transcription activation of Adr1-dependent genes. Mol. Microbiol. 62, 1433–1446. doi: 10.1111/j.1365-2958.2006.05451.x, PMID: 17121596

[ref9] Ahn I. Kang C. S. Han J. (2023). Where should siRNAs go: applicable organs for siRNA drugs. Exp. Mol. Med. 55, 1283–1292. doi: 10.1038/s12276-023-00998-y, PMID: 37430086 PMC10393947

[ref10] Al Aboud N. M. Tupper C. Jialal I. (2024). Genetics, epigenetic mechanism. Treasure Island, FL: StatPearls. 30422591

[ref11] Alaskhar Alhamwe B. Khalaila R. Wolf J. von Bülow V. Harb H. Alhamdan F.. (2018). Histone modifications and their role in epigenetics of atopy and allergic diseases. Allergy Asthma Clinic. Immunol. 14:39. doi: 10.1186/s13223-018-0259-4, PMID: 29796022 PMC5966915

[ref12] Alizadeh F. Javadi M. Karami A. A. Gholaminejad F. Kavianpour M. Haghighian H. K. (2018). Curcumin nanomicelle improves semen parameters, oxidative stress, inflammatory biomarkers, and reproductive hormones in infertile men: a randomized clinical trial. Phytotherapy Res. 32, 514–521. doi: 10.1002/ptr.5998, PMID: 29193350

[ref13] Alshaer W. Zureigat H. Al Karaki A. Al-Kadash A. Gharaibeh L. Hatmal M. M.. (2021). siRNA: mechanism of action, challenges, and therapeutic approaches. Eur. J. Pharmacol. 905:174178. doi: 10.1016/j.ejphar.2021.174178, PMID: 34044011

[ref14] Amini A. Khadivar P. Ahmadnia A. Alipour M. Majeed M. Jamialahmadi T.. (2021). Role of curcumin in regulating Long noncoding RNA expression in Cancer. Adv. Exp. Med. Biol. 1308, 13–23. doi: 10.1007/978-3-030-64872-5_2, PMID: 33861433

[ref15] Anand P. Kunnumakkara A. B. Newman R. A. Aggarwal B. B. (2007). Bioavailability of curcumin: problems and promises. Mol. Pharm. 4, 807–818. doi: 10.1021/mp700113r, PMID: 17999464

[ref16] Asadi S. Gholami M. S. Siassi F. Qorbani M. Khamoshian K. Sotoudeh G. (2019). Nano curcumin supplementation reduced the severity of diabetic sensorimotor polyneuropathy in patients with type 2 diabetes mellitus: a randomized double-blind placebo- controlled clinical trial. Complement. Ther. Med. 43, 253–260. doi: 10.1016/j.ctim.2019.02.014, PMID: 30935539

[ref17] Azzini E. Peña-Corona S. I. Hernández-Parra H. Chandran D. Saleena L. A. K. Sawikr Y.. (2024). Neuroprotective and anti-inflammatory effects of curcumin in Alzheimer's disease: targeting neuroinflammation strategies. Phytother. Res. 38, 3169–3189. doi: 10.1002/ptr.8200, PMID: 38616356

[ref18] Balusu S. Horré K. Thrupp N. Craessaerts K. Snellinx A. Serneels L.. (2023). MEG3 activates necroptosis in human neuron xenografts modeling Alzheimer's disease. Science 381, 1176–1182. doi: 10.1126/science.abp9556, PMID: 37708272 PMC7615236

[ref19] Banerjee S. Chakravarty A. R. (2015). Metal complexes of curcumin for cellular imaging, targeting, and photoinduced anticancer activity. Acc. Chem. Res. 48, 2075–2083. doi: 10.1021/acs.accounts.5b00127, PMID: 26158541

[ref20] Bannister A. J. Kouzarides T. (2011). Regulation of chromatin by histone modifications. Cell Res. 21, 381–395. doi: 10.1038/cr.2011.22, PMID: 21321607 PMC3193420

[ref21] Bararia A. Das A. Mitra S. Banerjee S. Chatterjee A. Sikdar N. (2023). Deoxyribonucleic acid methylation driven aberrations in pancreatic cancer-related pathways. World J. Gastroint. Oncol. 15, 1505–1519. doi: 10.4251/wjgo.v15.i9.1505, PMID: 37746645 PMC10514732

[ref22] Bhaskaran M. Mohan M. (2014). MicroRNAs: history, biogenesis, and their evolving role in animal development and disease. Vet. Pathol. 51, 759–774. doi: 10.1177/0300985813502820, PMID: 24045890 PMC4013251

[ref23] Bird A. (2002). DNA methylation patterns and epigenetic memory. Genes Dev. 16, 6–21. doi: 10.1101/gad.947102, PMID: 11782440

[ref24] Cai Z. Liang C. Huang K. Luo J. Lu R. Lai Y.. (2025). Curcumin prevents neurodegeneration by blocking HDAC6-NLRP3 pathway-dependent neuroinflammation in Parkinson's disease. Int. Immunopharmacol. 146:113928. doi: 10.1016/j.intimp.2024.113928, PMID: 39724731

[ref25] Cai J. Sun H. Zheng B. Xie M. Xu C. Zhang G.. (2021). Curcumin attenuates lncRNA H19-induced epithelial-mesenchymal transition in tamoxifen-resistant breast cancer cells. Mol. Med. Rep. 23:10. doi: 10.3892/mmr.2020.11651 33179087 PMC7673326

[ref26] Cao M. Duan Z. Wang X. Gong P. Zhang L. Ruan B. (2024). Curcumin promotes diabetic foot ulcer wound healing by inhibiting miR-152-3p and activating the FBN1/TGF-β pathway. Mol. Biotechnol. 66, 1266–1278. doi: 10.1007/s12033-023-01027-z, PMID: 38206528 PMC11087368

[ref27] Carroll R. E. Benya R. V. Turgeon D. K. Vareed S. Neuman M. Rodriguez L.. (2011). Phase IIa clinical trial of curcumin for the prevention of colorectal neoplasia. Cancer Prev. Res. (Phila.) 4, 354–364. doi: 10.1158/1940-6207.CAPR-10-0098, PMID: 21372035 PMC4136551

[ref28] Carthew R. W. Sontheimer E. J. (2009). Origins and mechanisms of miRNAs and siRNAs. Cell 136, 642–655. doi: 10.1016/j.cell.2009.01.035, PMID: 19239886 PMC2675692

[ref29] Chao L. Yang S. Li H. Long C. Xi Q. Zuo Y. (2022). Competitive binding of TET1 and DNMT3A/B cooperates the DNA methylation pattern in human embryonic stem cells. Biochim. Biophys. Acta 1865:194861. doi: 10.1016/j.bbagrm.2022.194861, PMID: 35998875

[ref30] Chen C. Li Y. Lu H. Liu K. Jiang W. Zhang Z.. (2022). Curcumin attenuates vascular calcification via the exosomal miR-92b-3p/KLF4 axis. Exp. Biol. Med. (Maywood) 247, 1420–1432. doi: 10.1177/15353702221095456, PMID: 35666058 PMC9493763

[ref31] Chen W. T. Wu H. T. Chang I. C. Chen H.-W. Fang W.-P. (2022). Preparation of curcumin-loaded liposome with high bioavailability by a novel method of high pressure processing. Chem. Phys. Lipids 244:105191. doi: 10.1016/j.chemphyslip.2022.105191, PMID: 35257749

[ref32] Chiu S. Terpstra K. J. Bureau Y. Hou J. Raheb H. Cernvosky Z.. (2013). Liposomal-formulated curcumin [Lipocurc™] targeting HDAC (histone deacetylase) prevents apoptosis and improves motor deficits in Park 7 (DJ-1)-knockout rat model of Parkinson's disease: implications for epigenetics-based nanotechnology-driven drug platform. J. Complement. Integr. Med. 10:20. doi: 10.1515/jcim-2013-0020 24200537

[ref33] Chiu S. S. Terpstra K. Woodbury-Farina M. Mishra R. Badmae V. Vaughese J.. (2020). Transforming curry extract to liposomal curcumin (LipocurcTM) in Parkinson disease (PD) therapeutics landscape: emerging role of epigenetics signaling and nanotechnology. EC Neurology. 12, 01–12.

[ref34] Chu F. Xue L. Miao H. (2020). Long noncoding RNA TP73-AS1 in human cancers. Clin. Chim. Acta 500, 104–108. doi: 10.1016/j.cca.2019.09.024, PMID: 31678571

[ref35] Cui C. Han Y. Li H. Yu H. Zhang B. Li G. (2022). Curcumin-driven reprogramming of the gut microbiota and metabolome ameliorates motor deficits and neuroinflammation in a mouse model of Parkinson’s disease. Front. Cell. Infect. Microbiol. 12:887407. doi: 10.3389/fcimb.2022.887407, PMID: 36034698 PMC9400544

[ref36] Cui Y. Song H. T. Zhang P. Yin X. Wang Y. Wei X.. (2022). Curcumin protects PC12 cells from a high glucose-induced inflammatory response by regulating the miR-218-5p/TLR4 axis. Medicine 101:e30967. doi: 10.1097/MD.0000000000030967, PMID: 36221434 PMC9543010

[ref37] Deaton A. M. Bird A. (2011). CpG islands and the regulation of transcription. Genes Dev. 25, 1010–1022. doi: 10.1101/gad.2037511, PMID: 21576262 PMC3093116

[ref38] Dehghani Z. Meratan A. A. Saboury A. A. Nemat-Gorgani M. (2020). Α-Synuclein fibrillation products trigger the release of hexokinase I from mitochondria: protection by curcumin, and possible role in pathogenesis of Parkinson's disease. Biochim. Biophys. Acta Biomembr. 1862:183251. doi: 10.1016/j.bbamem.2020.183251, PMID: 32113849

[ref39] Dekker F. J. Haisma H. J. (2009). Histone acetyl transferases as emerging drug targets. Drug Discov. Today 14, 942–948. doi: 10.1016/j.drudis.2009.06.008, PMID: 19577000

[ref40] Deng Y. Lu X. Wang L. Li T. Ding Y. Cao H.. (2014). Curcumin inhibits the AKT/NF-κB signaling via CpG demethylation of the promoter and restoration of NEP in the N2a cell line. AAPS J. 16, 649–657. doi: 10.1208/s12248-014-9605-8, PMID: 24756894 PMC4070258

[ref41] Ding Q. Gao Z. Chen K. Zhang Q. Hu S. Zhao L. (2022). Inflammation-related epigenetic modification: the bridge between immune and metabolism in type 2 diabetes. Front. Immunol. 13:883410. doi: 10.3389/fimmu.2022.883410, PMID: 35603204 PMC9120428

[ref42] Dong S. Zeng Q. Mitchell E. S. Xiu J. Duan Y. Li C.. (2012). Curcumin enhances neurogenesis and cognition in aged rats: implications for transcriptional interactions related to growth and synaptic plasticity. PLoS One 7:e31211. doi: 10.1371/journal.pone.0031211, PMID: 22359574 PMC3281036

[ref43] Du L. Xie Z. Wu L.-c. Chiu M. Lin J. Chan K. K.. (2012). Reactivation of RASSF1A in breast cancer cells by curcumin. Nutr. Cancer 64, 1228–1235. doi: 10.1080/01635581.2012.717682, PMID: 23145775 PMC5082258

[ref44] El Oirdi M. Farhan M. (2024). Clinical trial findings and drug development challenges for curcumin in infectious disease prevention and treatment. Life 14:1138. doi: 10.3390/life14091138, PMID: 39337921 PMC11432846

[ref45] Endo Y. Fujimoto M. Ito N. Takahashi Y. Kitago M. Gotoh M.. (2021). Clinicopathological impacts of DNA methylation alterations on pancreatic ductal adenocarcinoma: prediction of early recurrence based on genome-wide DNA methylation profiling. J. Cancer Res. Clin. Oncol. 147, 1341–1354. doi: 10.1007/s00432-021-03541-6, PMID: 33635431 PMC8021514

[ref46] Fang M. Z. Wang Y. Ai N. Hou Z. Sun Y. Lu H.. (2003). Tea polyphenol (−)-epigallocatechin-3-gallate inhibits DNA methyltransferase and reactivates methylation-silenced genes in cancer cell lines. Cancer Res. 63, 7563–7570. 14633667

[ref47] Farsetti A. Illi B. Gaetano C. (2023). How epigenetics impacts on human diseases. Eur. J. Intern. Med. 114, 15–22. doi: 10.1016/j.ejim.2023.05.036, PMID: 37277249

[ref48] Flora G. Gupta D. Tiwari A. (2012). Toxicity of lead: a review with recent updates. Interdiscip. Toxicol. 5, 47–58. doi: 10.2478/v10102-012-0009-2, PMID: 23118587 PMC3485653

[ref49] Friedrich M. Aigner A. (2022). Therapeutic siRNA: state-of-the-art and future perspectives. BioDrugs 36, 549–571. doi: 10.1007/s40259-022-00549-3, PMID: 35997897 PMC9396607

[ref50] Fu X. Zhang J. Huang X. Mo Z. Sang Z. Duan W.. (2021). Curcumin antagonizes glucose fluctuation-induced renal injury by inhibiting aerobic glycolysis via the miR-489/LDHA pathway. Mediat. Inflamm. 2021, 1–25. doi: 10.1155/2021/6104529 PMC8387199 34456629

[ref51] Fyodorov D. V. Zhou B. R. Skoultchi A. I. Bai Y. (2018). Emerging roles of linker histones in regulating chromatin structure and function. Nat. Rev. Mol. Cell Biol. 19, 192–206. doi: 10.1038/nrm.2017.94, PMID: 29018282 PMC5897046

[ref52] Gan L. Li C. Wang J. Guo X. (2016). Curcumin modulates the effect of histone modification on the expression of chemokines by type II alveolar epithelial cells in a rat COPD model. Int. J. Chron. Obstruct. Pulmon. Dis. 11, 2765–2773. doi: 10.2147/COPD.S113978, PMID: 27853364 PMC5106221

[ref53] Gansen A. Tóth K. Schwarz N. Langowski J. (2015). Opposing roles of H3- and H4-acetylation in the regulation of nucleosome structure––a FRET study. Nucleic Acids Res. 43, 1433–1443. doi: 10.1093/nar/gku1354, PMID: 25589544 PMC4330349

[ref54] Garcea G. Jones D. Singh R. Dennison A. Farmer P. Sharma R.. (2004). Detection of curcumin and its metabolites in hepatic tissue and portal blood of patients following oral administration. Br. J. Cancer 90, 1011–1015. doi: 10.1038/sj.bjc.6601623, PMID: 14997198 PMC2409622

[ref55] García-Niño W. R. Pedraza-Chaverrí J. (2014). Protective effect of curcumin against heavy metals-induced liver damage. Food Chem. Toxicol. 69, 182–201. doi: 10.1016/j.fct.2014.04.016, PMID: 24751969

[ref56] Geng H. H. Li R. Su Y. M. Xiao J. Pan M. Cai X. X.. (2016). Curcumin protects cardiac myocyte against hypoxia-induced apoptosis through upregulating miR-7a/b expression. Biomed. Pharmacother. 81, 258–264. doi: 10.1016/j.biopha.2016.04.020, PMID: 27261602

[ref57] Gera M. Sharma N. Ghosh M. Huynh D. L. Lee S. J. Min T.. (2017). Nanoformulations of curcumin: an emerging paradigm for improved remedial application. Oncotarget 8, 66680–66698. doi: 10.18632/oncotarget.19164, PMID: 29029547 PMC5630447

[ref58] Giordano S. Darley-Usmar V. Zhang J. (2014). Autophagy as an essential cellular antioxidant pathway in neurodegenerative disease. Redox Biol. 2, 82–90. doi: 10.1016/j.redox.2013.12.013, PMID: 24494187 PMC3909266

[ref59] Goel A. Kunnumakkara A. B. Aggarwal B. B. (2008). Curcumin as "Curecumin": from kitchen to clinic. Biochem. Pharmacol. 75, 787–809. doi: 10.1016/j.bcp.2007.08.016, PMID: 17900536

[ref60] Gong J. Sun D. (2022). Study on the mechanism of curcumin to reduce the inflammatory response of temporal lobe in Alzheimer's disease by regulating miR-146a. Minerva Med. 113, 109–118. doi: 10.23736/S0026-4806.20.06463-0, PMID: 32207596

[ref61] Gopalakrishnan S. Van Emburgh B. O. Robertson K. D. (2008). DNA methylation in development and human disease. Mutat. Res. 647, 30–38. doi: 10.1016/j.mrfmmm.2008.08.006, PMID: 18778722 PMC2647981

[ref62] Gupta S. C. Patchva S. Aggarwal B. B. (2013). Therapeutic roles of curcumin: lessons learned from clinical trials. AAPS J. 15, 195–218. doi: 10.1208/s12248-012-9432-8, PMID: 23143785 PMC3535097

[ref63] Halegoua-DeMarzio D. Navarro V. Ahmad J. Avula B. Barnhart H. Barritt A. S.. (2023). Liver injury associated with turmeric-a growing problem: ten cases from the drug-induced liver injury network [DILIN]. Am. J. Med. 136, 200–206. doi: 10.1016/j.amjmed.2022.09.026, PMID: 36252717 PMC9892270

[ref64] Harb H. Alashkar Alhamwe B. Garn H. Renz H. Potaczek D. P. (2016). Recent developments in epigenetics of pediatric asthma. Curr. Opin. Pediatr. 28, 754–763. doi: 10.1097/MOP.0000000000000424, PMID: 27662207

[ref65] Hassan F. U. Rehman M. S. Khan M. S. Ali M. A. Javed A. Nawaz A.. (2019). Curcumin as an alternative epigenetic modulator: mechanism of action and potential effects. Front. Genet. 10:514. doi: 10.3389/fgene.2019.00514, PMID: 31214247 PMC6557992

[ref66] Hassaninasab A. Hashimoto Y. Tomita-Yokotani K. Kobayashi M. (2011). Discovery of the curcumin metabolic pathway involving a unique enzyme in an intestinal microorganism. Proc. Natl. Acad. Sci. USA 108, 6615–6620. doi: 10.1073/pnas.1016217108, PMID: 21467222 PMC3080977

[ref67] He Y. Liu Y. Zhang M. (2025). The beneficial effects of curcumin on aging and age-related diseases: from oxidative stress to antioxidant mechanisms, brain health and apoptosis. Front. Aging Neurosci. 17:1533963. doi: 10.3389/fnagi.2025.1533963, PMID: 39906716 PMC11788355

[ref68] Healy S. Khan P. He S. Davie J. R. (2012). Histone H3 phosphorylation, immediate-early gene expression, and the nucleosomal response: a historical perspective. Biochem. Cell Biol. 90, 39–54. doi: 10.1139/o11-092, PMID: 22250664

[ref69] Hegde M. Girisa S. BharathwajChetty B. Vishwa R. Kunnumakkara A. B. (2023). Curcumin formulations for better bioavailability: what we learned from clinical trials thus far? ACS Omega 8, 10713–10746. doi: 10.1021/acsomega.2c07326, PMID: 37008131 PMC10061533

[ref70] Heidari H. Bagherniya M. Majeed M. Sathyapalan T. Jamialahmadi T. Sahebkar A. (2023). Curcumin-piperine co-supplementation and human health: a comprehensive review of preclinical and clinical studies. Phytother. Res. 37, 1462–1487. doi: 10.1002/ptr.7737, PMID: 36720711

[ref71] Henrotin Y. Clutterbuck A. L. Allaway D. Lodwig E. M. Harris P. Mathy-Hartert M.. (2010). Biological actions of curcumin on articular chondrocytes. Osteoarthr. Cartil. 18, 141–149. doi: 10.1016/j.joca.2009.10.002, PMID: 19836480

[ref72] Hoehle S. I. Pfeiffer E. Metzler M. (2007). Glucuronidation of curcuminoids by human microsomal and recombinant UDP-glucuronosyltransferases. Mol. Nutr. Food Res. 51, 932–938. doi: 10.1002/mnfr.200600283, PMID: 17628876

[ref73] Hoehle S. I. Pfeiffer E. Sólyom A. M. Metzler M. (2006). Metabolism of curcuminoids in tissue slices and subcellular fractions from rat liver. J. Agric. Food Chem. 54, 756–764. doi: 10.1021/jf058146a, PMID: 16448179

[ref74] Holley R. W. Apgar J. Everett G. A. Madison J. T. Marquisee M. Merrill S. H.. (1965). STRUCTURE OF A RIBONUCLEIC ACID. Science 147, 1462–1465. doi: 10.1126/science.147.3664.1462, PMID: 14263761

[ref75] Hu S. Belcaro G. Dugall M. Peterzan P. Hosoi M. Ledda A.. (2018). Interaction study between antiplatelet agents, anticoagulants, thyroid replacement therapy and a bioavailable formulation of curcumin (Meriva®). Eur. Rev. Med. Pharmacol. Sci. 22, 5042–5046. doi: 10.26355/eurrev_201808_15647 30070343

[ref76] Hu J. Shen T. Xie J. Wang S. He Y. Zhu F. (2017). Curcumin modulates covalent histone modification and TIMP1 gene activation to protect against vascular injury in a hypertension rat model. Exp. Ther. Med. 14, 5896–5902. doi: 10.3892/etm.2017.5318, PMID: 29285138 PMC5740590

[ref77] Huai B. Huang C. Hu L. (2022). Curcumin suppresses TGF-β2-induced proliferation, migration, and invasion in Lens epithelial cells by targeting KCNQ1OT1/miR-377-3p/COL1A2 Axis in posterior capsule opacification. Curr. Eye Res. 47, 715–726. doi: 10.1080/02713683.2021.2021537, PMID: 35179079

[ref78] Huang H. C. Chang P. Lu S. Y. Zheng B. W. Jiang Z. F. (2015). Protection of curcumin against amyloid-β-induced cell damage and death involves the prevention from NMDA receptor-mediated intracellular Ca2+ elevation. J. Recept. Signal Transduct. Res. 35, 450–457. doi: 10.3109/10799893.2015.1006331, PMID: 26053510

[ref79] Huang H. C. Tang D. Xu K. Jiang Z. F. (2014). Curcumin attenuates amyloid-β-induced tau hyperphosphorylation in human neuroblastoma SH-SY5Y cells involving PTEN/Akt/GSK-3β signaling pathway. J. Recept. Signal Transduct. Res. 34, 26–37. doi: 10.3109/10799893.2013.848891, PMID: 24188406

[ref80] Huang H. C. Xu K. Jiang Z. F. (2012). Curcumin-mediated neuroprotection against amyloid-β-induced mitochondrial dysfunction involves the inhibition of GSK-3β. J Alzheimer's Dis 32, 981–996. doi: 10.3233/JAD-2012-120688, PMID: 22886017

[ref81] Hussain Y. Alam W. Ullah H. Dacrema M. Daglia M. Khan H.. (2022). Antimicrobial potential of curcumin: therapeutic potential and challenges to clinical applications. Antibiotics 11:322. doi: 10.3390/antibiotics11030322, PMID: 35326785 PMC8944843

[ref82] Izadi M. Sadri N. Abdi A. Zadeh M. M. R. Jalaei D. Ghazimoradi M. M.. (2024). Longevity and anti-aging effects of curcumin supplementation. GeroScience 46, 2933–2950. doi: 10.1007/s11357-024-01092-5, PMID: 38409646 PMC11009219

[ref83] Jakubczyk K. Drużga A. Katarzyna J. Skonieczna-Żydecka K. (2020). Antioxidant potential of curcumin-a meta-analysis of randomized clinical trials. Antioxidants 9:92. doi: 10.3390/antiox9111092, PMID: 33172016 PMC7694612

[ref84] Jiang L. Li W. Gong X. L. Wang G. Y. Zhao F. Han L. (2024). Curcumin alleviates myocardial inflammation, apoptosis, and oxidative stress induced by acute pulmonary embolism by regulating microRNA-145-5P/insulin receptor substrate 1 axis. J. Physiol. Pharmacol. 75:3. doi: 10.26402/jpp.2024.1.03 38583436

[ref85] Jiang T. F. Zhang Y. J. Zhou H. Y. Wang H. M. Tian L. P. Liu J.. (2013). Curcumin ameliorates the neurodegenerative pathology in A53T α-synuclein cell model of Parkinson's disease through the downregulation of mTOR/p70S6K signaling and the recovery of macroautophagy. J. Neuroimmune Pharmacol. 8, 356–369. doi: 10.1007/s11481-012-9431-7, PMID: 23325107

[ref86] Jiao Y. Wilkinson J. Di X. Wang W. Hatcher H. Kock N. D.. (2009). Curcumin, a cancer chemopreventive and chemotherapeutic agent, is a biologically active iron chelator. Blood 113, 462–469. doi: 10.1182/blood-2008-05-155952, PMID: 18815282 PMC2615657

[ref87] Jin B. Tao Q. Peng J. Soo H. M. Wu W. Ying J.. (2008). DNA methyltransferase 3B (DNMT3B) mutations in ICF syndrome lead to altered epigenetic modifications and aberrant expression of genes regulating development, neurogenesis and immune function. Hum. Mol. Genet. 17, 690–709. doi: 10.1093/hmg/ddm341, PMID: 18029387

[ref88] Jin B. Yao B. Li J. L. Fields C. R. Delmas A. L. Liu C.. (2009). DNMT1 and DNMT3B modulate distinct polycomb-mediated histone modifications in colon cancer. Cancer Res. 69, 7412–7421. doi: 10.1158/0008-5472.CAN-09-0116, PMID: 19723660 PMC2745494

[ref89] Kakkar V. Kumari P. Kaur J. Chholta S. (2023). Curcumin Nanoformulations in Neurodegenerative Diseases. In: Rai, M., Feitosa, C.M. (eds) Curcumin and Neurodegenerative Diseases. (Singapore: Springer). 379–402. doi: 10.1007/978-981-99-7731-4_18

[ref90] Keihanian F. Saeidinia A. Bagheri R. K. Johnston T. P. Sahebkar A. (2018). Curcumin, hemostasis, thrombosis, and coagulation. J. Cell. Physiol. 233, 4497–4511. doi: 10.1002/jcp.26249, PMID: 29052850

[ref91] Khayatan D. Razavi S. M. Arab Z. N. Nasoori H. Fouladi A. Pasha A. V. K.. (2025). Targeting mTOR with curcumin: therapeutic implications for complex diseases. Inflammopharmacology 33, 1583–1616. doi: 10.1007/s10787-025-01643-y, PMID: 39955697

[ref92] Khezri K. Saeedi M. Mohammadamini H. Zakaryaei A. S. (2021). A comprehensive review of the therapeutic potential of curcumin nanoformulations. Phytother. Res. 35, 5527–5563. doi: 10.1002/ptr.7190, PMID: 34131980

[ref93] Kim D. C. Ku S. K. Bae J. S. (2012). Anticoagulant activities of curcumin and its derivative. BMB Rep. 45, 221–226. doi: 10.5483/bmbrep.2012.45.4.221, PMID: 22531131

[ref94] Kocaadam B. Şanlier N. (2017). Curcumin, an active component of turmeric (*Curcuma longa*), and its effects on health. Crit. Rev. Food Sci. Nutr. 57, 2889–2895. doi: 10.1080/10408398.2015.1077195, PMID: 26528921

[ref95] Kumar V. Kesharwani R. Patel D. K. Verma A. Mehanna M. G. Mohammad A.. (2024). Epigenetic impact of curcumin and Thymoquinone on Cancer therapeutics. Curr. Med. Chem. 32, 2183–2201. doi: 10.2174/0109298673288542240327112351, PMID: 38584537

[ref96] Kundu S. Ji F. Sunwoo H. Jain G. Lee J. T. Sadreyev R. I.. (2017). Polycomb repressive complex 1 generates discrete compacted domains that change during differentiation. Mol. Cell 65, 432–446.e5. e5. doi: 10.1016/j.molcel.2017.01.009, PMID: 28157505 PMC5421375

[ref97] Kurdistani S. K. Tavazoie S. Grunstein M. (2004). Mapping global histone acetylation patterns to gene expression. Cell 117, 721–733. doi: 10.1016/j.cell.2004.05.023, PMID: 15186774

[ref98] Kuszewski J. C. Wong R. H. X. Howe P. R. C. (2018). Can curcumin counteract cognitive decline? Clinical trial evidence and rationale for combining ω-3 fatty acids with curcumin. Adv. Nutr. 9, 105–113. doi: 10.1093/advances/nmx013, PMID: 29659685 PMC5916424

[ref99] Lewinska A. Wnuk M. Grabowska W. Zabek T. Semik E. Sikora E.. (2015). Curcumin induces oxidation-dependent cell cycle arrest mediated by SIRT7 inhibition of rDNA transcription in human aortic smooth muscle cells. Toxicol. Lett. 233, 227–238. doi: 10.1016/j.toxlet.2015.01.019, PMID: 25644192

[ref100] Li E. (2002). Chromatin modification and epigenetic reprogramming in mammalian development. Nat. Rev. Genet. 3, 662–673. doi: 10.1038/nrg887, PMID: 12209141

[ref101] Li E. Bestor T. H. Jaenisch R. (1992). Targeted mutation of the DNA methyltransferase gene results in embryonic lethality. Cell 69, 915–926. doi: 10.1016/0092-8674(92)90611-F, PMID: 1606615

[ref102] Li G. Bu J. Zhu Y. Xiao X. Liang Z. Zhang R. (2015). Curcumin improves bone microarchitecture in glucocorticoid-induced secondary osteoporosis mice through the activation of microRNA-365 via regulating MMP-9. Int. J. Clin. Exp. Pathol. 8, 15684–15695. 26884838 PMC4730051

[ref103] Li J. Jiang X. Li Z. Huang L. Zhou Y. Liu Y.. (2019). Long noncoding RNA GHET1 in human cancer. Clin. Chim. Acta 488, 111–115. doi: 10.1016/j.cca.2018.11.007, PMID: 30399371

[ref104] Li D. Lu Z. Jia J. Zheng Z. Lin S. (2013). Curcumin ameliorates podocytic adhesive capacity damage under mechanical stress by inhibiting miR-124 expression. Kidney Blood Press. Res. 38, 61–71. doi: 10.1159/000355755, PMID: 24556741

[ref105] Li J. Wang S. Zhang S. Cheng D. Yang X. Wang Y.. (2021). Curcumin slows the progression of Alzheimer's disease by modulating mitochondrial stress responses via JMJD3-H3K27me3-BDNF axis. Am. J. Transl. Res. 13, 13380–13393. 35035682 PMC8748089

[ref106] Li Z. Zhang Z. Ren Y. Wang Y. Fang J. Yue H.. (2021). Aging and age-related diseases: from mechanisms to therapeutic strategies. Biogerontology 22, 165–187. doi: 10.1007/s10522-021-09910-5, PMID: 33502634 PMC7838467

[ref107] Li X. Zhu R. Jiang H. Yin Q. Gu J. Chen J.. (2022a). Autophagy enhanced by curcumin ameliorates inflammation in atherogenesis via the TFEB-P300-BRD4 axis. Acta Pharm. Sin. B 12, 2280–2299. doi: 10.1016/j.apsb.2021.12.014, PMID: 35646539 PMC9136579

[ref108] Li X. Zhu J. Zhong Y. Liu C. Yao M. Sun Y.. (2022b). Targeting long noncoding RNA-AQP4-AS1 for the treatment of retinal neurovascular dysfunction in diabetes mellitus. EBioMedicine 77:103857. doi: 10.1016/j.ebiom.2022.103857, PMID: 35172268 PMC8850682

[ref109] Lietz C. E. Newman E. T. Kelly A. D. Xiang D. H. Zhang Z. Luscko C. A.. (2022). Genome-wide DNA methylation patterns reveal clinically relevant predictive and prognostic subtypes in human osteosarcoma. Communic. Biol. 5:213. doi: 10.1038/s42003-022-03117-1, PMID: 35260776 PMC8904843

[ref110] Lister R. Pelizzola M. Dowen R. H. Hawkins R. D. Hon G. Tonti-Filippini J.. (2009). Human DNA methylomes at base resolution show widespread epigenomic differences. Nature 462, 315–322. doi: 10.1038/nature08514, PMID: 19829295 PMC2857523

[ref111] Liu H. Y. Fu X. Li Y. F. Li X. L. Ma Z. Y. Zhang Y.. (2019). miR-15b-5p targeting amyloid precursor protein is involved in the anti-amyloid eflect of curcumin in swAPP695-HEK293 cells. Neural Regen. Res. 14, 1603–1609. doi: 10.4103/1673-5374.255979, PMID: 31089060 PMC6557094

[ref112] Liu S. Liu J. He L. Liu L. Cheng B. Zhou F.. (2022). A comprehensive review on the benefits and problems of curcumin with respect to human health. Molecules 27:400. doi: 10.3390/molecules27144400, PMID: 35889273 PMC9319031

[ref113] Liu Y. Sun Y. Yang J. Wu D. Yu S. Liu J.. (2024). DNMT1-targeting remodeling global DNA hypomethylation for enhanced tumor suppression and circumvented toxicity in oral squamous cell carcinoma. Mol. Cancer 23:104. doi: 10.1186/s12943-024-01993-1, PMID: 38755637 PMC11097543

[ref114] Liu G. Xiang T. Wu Q. F. Wang W. X. (2016). Curcumin suppresses the proliferation of gastric cancer cells by downregulating H19. Oncol. Lett. 12, 5156–5162. doi: 10.3892/ol.2016.5354, PMID: 28105222 PMC5228417

[ref115] Liu Z. Xie Z. Jones W. Pavlovicz R. E. Liu S. Yu J.. (2009). Curcumin is a potent DNA hypomethylation agent. Bioorg. Med. Chem. Lett. 19, 706–709. doi: 10.1016/j.bmcl.2008.12.041, PMID: 19112019

[ref116] López-Otín C. Blasco M. A. Partridge L. Serrano M. Kroemer G. (2013). The hallmarks of aging. Cell 153, 1194–1217. doi: 10.1016/j.cell.2013.05.039, PMID: 23746838 PMC3836174

[ref117] Lopresti A. L. (2018). The problem of curcumin and its bioavailability: could its gastrointestinal influence contribute to its overall health-enhancing effects? Adv. Nutr. 9, 41–50. doi: 10.1093/advances/nmx011, PMID: 29438458 PMC6333932

[ref118] Lorenzi L. Avila Cobos F. Decock A. Everaert C. Helsmoortel H. Lefever S.. (2019). Long noncoding RNA expression profiling in cancer: challenges and opportunities. Genes Chromosomes Cancer 58, 191–199. doi: 10.1002/gcc.22709, PMID: 30461116

[ref119] Lorincz M. C. Dickerson D. R. Schmitt M. Groudine M. (2004). Intragenic DNA methylation alters chromatin structure and elongation efficiency in mammalian cells. Nat. Struct. Mol. Biol. 11, 1068–1075. doi: 10.1038/nsmb840, PMID: 15467727

[ref120] Lozano-Vidal N. Bink D. I. Boon R. A. (2019). Long noncoding RNA in cardiac aging and disease. J. Mol. Cell Biol. 11, 860–867. doi: 10.1093/jmcb/mjz046, PMID: 31152659 PMC6884711

[ref121] Lu T. X. Rothenberg M. E. (2018). MicroRNA. J. Allergy Clin. Immunol. 141, 1202–1207. doi: 10.1016/j.jaci.2017.08.034, PMID: 29074454 PMC5889965

[ref122] Lv H. Wang Y. Yang X. Ling G. Zhang P. (2023). Application of curcumin nanoformulations in Alzheimer’s disease: prevention, diagnosis and treatment. Nutr. Neurosci. 26, 727–742. doi: 10.1080/1028415X.2022.2084550, PMID: 35694842

[ref123] Mahmoudi Z. Jahani M. Nekouian R. (2023). Role of curcumin on miR-26a and its effect on DNMT1, DNMT3b, and MEG3 expression in A549 lung cancer cell. J. Cancer Res. Ther. 19, 1788–1793. doi: 10.4103/jcrt.jcrt_2181_21, PMID: 38376279

[ref124] Maleki Dizaj S. Alipour M. Dalir Abdolahinia E. Ahmadian E. Eftekhari A. Forouhandeh H.. (2022). Curcumin nanoformulations: beneficial nanomedicine against cancer. Phytother. Res. 36, 1156–1181. doi: 10.1002/ptr.7389, PMID: 35129230

[ref125] Marczylo T. H. Steward W. P. Gescher A. J. (2009). Rapid analysis of curcumin and curcumin metabolites in rat biomatrices using a novel ultraperformance liquid chromatography (UPLC) method. J. Agric. Food Chem. 57, 797–803. doi: 10.1021/jf803038f, PMID: 19152267

[ref126] Maugeri A. Mazzone M. G. Giuliano F. Vinciguerra M. Basile G. Barchitta M.. (2018). Curcumin modulates DNA methyltransferase functions in a cellular model of diabetic retinopathy. Oxidative Med. Cell. Longev. 2018:5407482. doi: 10.1155/2018/5407482, PMID: 30057682 PMC6051042

[ref127] Mei X. Xu D. Xu S. Zheng Y. Xu S. (2011). Gastroprotective and antidepressant effects of a new zinc(II)-curcumin complex in rodent models of gastric ulcer and depression induced by stresses. Pharmacol. Biochem. Behav. 99, 66–74. doi: 10.1016/j.pbb.2011.04.002, PMID: 21513730

[ref128] Meja K. K. Rajendrasozhan S. Adenuga D. Biswas S. K. Sundar I. K. Spooner G.. (2008). Curcumin restores corticosteroid function in monocytes exposed to oxidants by maintaining HDAC2. Am. J. Respir. Cell Mol. Biol. 39, 312–323. doi: 10.1165/rcmb.2008-0012OC, PMID: 18421014 PMC2542449

[ref129] Menon V. P. Sudheer A. R. (2007). Antioxidant and anti-inflammatory properties of curcumin. Adv. Exp. Med. Biol. 595, 105–125. doi: 10.1007/978-0-387-46401-5_3 17569207

[ref130] Messerschmidt D. M. Knowles B. B. Solter D. (2014). DNA methylation dynamics during epigenetic reprogramming in the germline and preimplantation embryos. Genes Dev. 28, 812–828. doi: 10.1101/gad.234294.113, PMID: 24736841 PMC4003274

[ref131] Montero-Hidalgo A. J. Jiménez-Vacas J. M. Gómez-Gómez E. Porcel-Pastrana F. Sáez-Martínez P. Pérez-Gómez J. M.. (2024). SRSF6 modulates histone-chaperone HIRA splicing to orchestrate AR and E2F activity in prostate cancer. Sci. Adv. 10:eado8231. doi: 10.1126/sciadv.ado8231, PMID: 39356765 PMC11446284

[ref132] Morera L. Lübbert M. Jung M. (2016). Targeting histone methyltransferases and demethylases in clinical trials for cancer therapy. Clin. Epigenetics 8:57. doi: 10.1186/s13148-016-0223-4, PMID: 27222667 PMC4877953

[ref133] Morimoto T. Sunagawa Y. Kawamura T. Takaya T. Wada H. Nagasawa A.. (2008). The dietary compound curcumin inhibits p300 histone acetyltransferase activity and prevents heart failure in rats. J. Clin. Invest. 118, 868–878. doi: 10.1172/JCI33160 18292809 PMC2248328

[ref134] Ms S. A. B. Waldman Ph D. H. Krings Ph D. B. Lamberth Ph D. J. Smith Ph D. J. McAllister Ph D. M. (2020). Effect of curcumin supplementation on exercise-induced oxidative stress, inflammation, muscle damage, and muscle soreness. J. Dietary Suppl. 17, 401–414. doi: 10.1080/19390211.2019.1604604, PMID: 31025894

[ref135] Mukwevho E. Matumba M. G. (2019). The influence of neonatal intake of curcumin on expression of genes associated with lipid metabolism and inflammatory cytokines: implication on obesity. FASEB J. 33:14. doi: 10.1096/fasebj.2019.33.1_supplement.487.14, PMID: 33414642

[ref136] Muta K. Obata Y. Oka S. Abe S. Minami K. Kitamura M.. (2016). Curcumin ameliorates nephrosclerosis via suppression of histone acetylation independent of hypertension. Nephrol. Dial. Transpl. 31, 1615–1623. doi: 10.1093/ndt/gfw036 27190365

[ref137] Naponelli V. Ramazzina I. Lenzi C. Bettuzzi S. Rizzi F. (2017). Green tea catechins for prostate cancer prevention: present achievements and future challenges. Antioxidants 6:26. doi: 10.3390/antiox6020026, PMID: 28379200 PMC5488006

[ref138] Naser A. F. A. Aziz W. M. Ahmed Y. R. Khalil W. K. B. Hamed M. A. A. (2022). Parkinsonism-like disease induced by rotenone in rats: treatment role of curcumin, dopamine agonist and adenosine a(2A) receptor antagonist. Curr. Aging Sci. 15, 65–76. doi: 10.2174/1874609814666210526115740, PMID: 34042043

[ref139] Nebrisi E. E. (2021). Neuroprotective activities of curcumin in Parkinson’s disease: a review of the literature. Int. J. Mol. Sci. 22:11248. doi: 10.3390/ijms222011248, PMID: 34681908 PMC8537234

[ref140] Neganova M. E. Klochkov S. G. Aleksandrova Y. R. Aliev G. (2022). Histone modifications in epigenetic regulation of cancer: perspectives and achieved progress. Semin. Cancer Biol. 83, 452–471. doi: 10.1016/j.semcancer.2020.07.015, PMID: 32814115

[ref141] Nehra S. Bhardwaj V. Ganju L. Saraswat D. (2015). Nanocurcumin prevents hypoxia induced stress in primary human ventricular cardiomyocytes by maintaining mitochondrial homeostasis. PLoS One 10:e0139121. doi: 10.1371/journal.pone.0139121, PMID: 26406246 PMC4583454

[ref142] Nelson K. M. Dahlin J. L. Bisson J. Graham J. Pauli G. F. Walters M. A. (2017). The essential medicinal chemistry of curcumin. J. Med. Chem. 60, 1620–1637. doi: 10.1021/acs.jmedchem.6b00975, PMID: 28074653 PMC5346970

[ref143] Nemeth K. Bayraktar R. Ferracin M. Calin G. A. (2024). Non-coding RNAs in disease: from mechanisms to therapeutics. Nat. Rev. Genet. 25, 211–232. doi: 10.1038/s41576-023-00662-1, PMID: 37968332

[ref144] Nervi C. Grignani F. (2014). RARs and microRNAs. Subcell. Biochem. 70, 151–179. doi: 10.1007/978-94-017-9050-5_8, PMID: 24962885

[ref145] Oguzturk H. Ciftci O. Aydin M. Timurkaan N. Beytur A. Yilmaz F. (2012). Ameliorative effects of curcumin against acute cadmium toxicity on male reproductive system in rats. Andrologia 44, 243–249. doi: 10.1111/j.1439-0272.2012.01273.x, PMID: 22257170

[ref146] Okano M. Bell D. W. Haber D. A. Li E. (1999). DNA methyltransferases Dnmt3a and Dnmt3b are essential for de novo methylation and mammalian development. Cell 99, 247–257. doi: 10.1016/S0092-8674(00)81656-6, PMID: 10555141

[ref147] Onoguchi-Mizutani R. Akimitsu N. (2022). Long noncoding RNA and phase separation in cellular stress response. J. Biochem. 171, 269–276. doi: 10.1093/jb/mvab156, PMID: 35080597

[ref148] Onufriev A. V. Schiessel H. (2019). The nucleosome: from structure to function through physics. Curr. Opin. Struct. Biol. 56, 119–130. doi: 10.1016/j.sbi.2018.11.003, PMID: 30710748

[ref149] Ouyang S. Zhang O. Xiang H. Yao Y. H. Fang Z. Y. (2022). Curcumin improves atherosclerosis by inhibiting the epigenetic repression of lncRNA MIAT to miR-124. Vascular 30, 1213–1223. doi: 10.1177/17085381211040974, PMID: 34989253

[ref150] Patil V. M. Das S. Balasubramanian K. (2016). Quantum chemical and docking insights into bioavailability enhancement of curcumin by piperine in pepper. J. Phys. Chem. A 120, 3643–3653. doi: 10.1021/acs.jpca.6b01434, PMID: 27111639

[ref151] Pawar K. S. Mastud R. N. Pawar S. K. Pawar S. S. Bhoite R. R. Bhoite R. R.. (2021). Oral curcumin with piperine as adjuvant therapy for the treatment of COVID-19: a randomized clinical trial. Front. Pharmacol. 12:669362. doi: 10.3389/fphar.2021.669362, PMID: 34122090 PMC8193734

[ref152] Payton F. Sandusky P. Alworth W. L. (2007). NMR study of the solution structure of curcumin. J. Nat. Prod. 70, 143–146. doi: 10.1021/np060263s, PMID: 17315954

[ref153] Pires S. F. de Barros J. S. da Costa S. S. de Oliveira Scliar M. van Helvoort Lengert A. Boldrini E.. (2023). DNA methylation patterns suggest the involvement of DNMT3B and TET1 in osteosarcoma development. Mol. Gen. Genomics. 298, 721–733. doi: 10.1007/s00438-023-02010-8 37020053

[ref154] Pivari F. Mingione A. Piazzini G. Ceccarani C. Ottaviano E. Brasacchio C.. (2022). Curcumin supplementation (Meriva®) modulates inflammation, lipid peroxidation and gut microbiota composition in chronic kidney disease. Nutrients 14:231. doi: 10.3390/nu14010231, PMID: 35011106 PMC8747135

[ref155] Plewka P. Raczynska K. D. (2022). Long intergenic noncoding RNAs affect biological pathways underlying autoimmune and neurodegenerative disorders. Mol. Neurobiol. 59, 5785–5808. doi: 10.1007/s12035-022-02941-0, PMID: 35796900 PMC9395482

[ref156] Poepsel S. Kasinath V. Nogales E. (2018). Cryo-EM structures of PRC2 simultaneously engaged with two functionally distinct nucleosomes. Nat. Struct. Mol. Biol. 25, 154–162. doi: 10.1038/s41594-018-0023-y, PMID: 29379173 PMC5805599

[ref157] Potaczek D. P. Harb H. Michel S. Alhamwe B. A. Renz H. Tost J. (2017). Epigenetics and allergy: from basic mechanisms to clinical applications. Epigenomics 9, 539–571. doi: 10.2217/epi-2016-0162, PMID: 28322581

[ref158] Pozniak T. Shcharbin D. Bryszewska M. (2022). Circulating microRNAs in medicine. Int. J. Mol. Sci. 23:996. doi: 10.3390/ijms23073996, PMID: 35409354 PMC8999557

[ref159] Prasad S. Gupta S. C. Tyagi A. K. Aggarwal B. B. (2014). Curcumin, a component of golden spice: from bedside to bench and back. Biotechnol. Adv. 32, 1053–1064. doi: 10.1016/j.biotechadv.2014.04.004, PMID: 24793420

[ref160] Prasanth M. I. Sivamaruthi B. S. Cheong C. S. Y. Verma K. Tencomnao T. Brimson J. M.. (2024). Role of epigenetic modulation in neurodegenerative diseases: implications of phytochemical interventions. Antioxidants 13:606. doi: 10.3390/antiox13050606, PMID: 38790711 PMC11118909

[ref161] Priyadarsini K. I. (2009). Photophysics, photochemistry and photobiology of curcumin: studies from organic solutions, bio-mimetics and living cells. J Photochem Photobiol C: Photochem Rev 10, 81–95. doi: 10.1016/j.jphotochemrev.2009.05.001

[ref162] Priyadarsini K. I. (2014). The chemistry of curcumin: from extraction to therapeutic agent. Molecules 19, 20091–20112. doi: 10.3390/molecules191220091, PMID: 25470276 PMC6270789

[ref163] Pucci D. Bellini T. Crispini A. D'Agnano I. Liguori P. F. Garcia-Orduna P.. (2012). DNA binding and cytotoxicity of fluorescent curcumin-based Zn (II) complexes. Med. Chem. Comm. 3, 462–468. doi: 10.1039/c2md00261b

[ref164] Qin L. Qiao C. Sheen V. Wang Y. Lu J. (2021). DNMT3L promotes neural differentiation by enhancing STAT1 and STAT3 phosphorylation independent of DNA methylation. Prog. Neurobiol. 201:102028. doi: 10.1016/j.pneurobio.2021.102028, PMID: 33636226

[ref165] Qin W. Zhu W. Sauter E. (2005). Resveratrol induced DNA methylation in ER+ breast cancer. Cancer Res. 65:647.

[ref166] Qiu B. Xu X. Yi P. Hao Y. (2020). Curcumin reinforces MSC-derived exosomes in attenuating osteoarthritis via modulating the miR-124/NF-kB and miR-143/ROCK1/TLR9 signalling pathways. J. Cell. Mol. Med. 24, 10855–10865. doi: 10.1111/jcmm.15714, PMID: 32776418 PMC7521270

[ref167] Rafiyan M. Mojtahedi H. (2025). Role of neuroinflammation in Alzheimer’s disease (AD). Immunol. Genet. J. 8, 150–188. doi: 10.18502/igj.v8i2.17998

[ref168] Rafiyan M. Sadeghmousavi S. Akbarzadehmoallemkolaei M. Rezaei N. (2023). Experimental animal models of chronic inflammation. Curr. Res. Immunol. 4:100063. doi: 10.1016/j.crimmu.2023.100063, PMID: 37334102 PMC10276141

[ref169] Reddy P. H. Manczak M. Yin X. Grady M. C. Mitchell A. Kandimalla R.. (2016). Protective effects of a natural product, curcumin, against amyloid β induced mitochondrial and synaptic toxicities in Alzheimer's disease. J. Investig. Med. 64, 1220–1234. doi: 10.1136/jim-2016-000240, PMID: 27521081 PMC5256118

[ref170] Remmerie A. Scott C. L. (2018). Macrophages and lipid metabolism. Cell. Immunol. 330, 27–42. doi: 10.1016/j.cellimm.2018.01.020, PMID: 29429624 PMC6108423

[ref171] Reuter S. Gupta S. C. Park B. Goel A. Aggarwal B. B. (2011). Epigenetic changes induced by curcumin and other natural compounds. Genes Nutr. 6, 93–108. doi: 10.1007/s12263-011-0222-1, PMID: 21516481 PMC3092901

[ref172] Riggs A. D. Xiong Z. (2004). Methylation and epigenetic fidelity. Proc. Natl. Acad. Sci. USA 101, 4–5. doi: 10.1073/pnas.0307781100, PMID: 14695893 PMC314126

[ref173] Robertson K. D. (2005). DNA methylation and human disease. Nat. Rev. Genet. 6, 597–610. doi: 10.1038/nrg1655, PMID: 16136652

[ref174] Rossetto D. Avvakumov N. Côté J. (2012). Histone phosphorylation: a chromatin modification involved in diverse nuclear events. Epigenetics 7, 1098–1108. doi: 10.4161/epi.21975, PMID: 22948226 PMC3469451

[ref175] Ruan Y. Xiong Y. Fang W. Yu Q. Mai Y. Cao Z.. (2022). Highly sensitive curcumin-conjugated nanotheranostic platform for detecting amyloid-beta plaques by magnetic resonance imaging and reversing cognitive deficits of Alzheimer's disease via NLRP3-inhibition. J. Nanobiotechnol. 20:322. doi: 10.1186/s12951-022-01524-4, PMID: 35836190 PMC9281113

[ref176] Saadati S. Sadeghi A. Mansour A. Yari Z. Poustchi H. Hedayati M.. (2019). Curcumin and inflammation in non-alcoholic fatty liver disease: a randomized, placebo controlled clinical trial. BMC Gastroenterol. 19:133. doi: 10.1186/s12876-019-1055-4, PMID: 31345163 PMC6659284

[ref177] Sabouni N. Marzouni H. Z. Palizban S. Meidaninikjeh S. Kesharwani P. Jamialahmadi T.. (2023). Role of curcumin and its nanoformulations in the treatment of neurological diseases through the effects on stem cells. J. Drug Target. 31, 243–260. doi: 10.1080/1061186X.2022.2141755, PMID: 36305097

[ref178] Saliminejad K. Khorram Khorshid H. R. Soleymani Fard S. Ghaffari S. H. (2019). An overview of microRNAs: biology, functions, therapeutics, and analysis methods. J. Cell. Physiol. 234, 5451–5465. doi: 10.1002/jcp.27486, PMID: 30471116

[ref179] Sanati M. Afshari A. R. Kesharwani P. Sahebkar A. (2023). Recent advances in codelivery of curcumin and siRNA as anticancer therapeutics. Eur. Polym. J. 198:112444. doi: 10.1016/j.eurpolymj.2023.112444

[ref180] Sang Q. Liu X. Wang L. Qi L. Sun W. Wang W.. (2018). Curcumin protects an SH-SY5Y cell model of Parkinson's disease against toxic injury by regulating HSP90. Cell. Physiol. Biochem. 51, 681–691. doi: 10.1159/000495326, PMID: 30463061

[ref181] Santana D. A. Smith M. A. C. Chen E. S. (2023). Histone modifications in Alzheimer's disease. Genes 14:347. doi: 10.3390/genes14020347, PMID: 36833274 PMC9956192

[ref182] Sato A. Kudo C. Yamakoshi H. Uehara Y. Ohori H. Ishioka C.. (2011). Curcumin analog GO-Y030 is a novel inhibitor of IKKβ that suppresses NF-κB signaling and induces apoptosis. Cancer Sci. 102, 1045–1051. doi: 10.1111/j.1349-7006.2011.01886.x, PMID: 21272158

[ref183] Saul D. Kosinsky R. L. (2021). Epigenetics of aging and aging-associated diseases. Int. J. Mol. Sci. 22:401. doi: 10.3390/ijms22010401, PMID: 33401659 PMC7794926

[ref184] Sawicka A. Hartl D. Goiser M. Pusch O. Stocsits R. R. Tamir I. M.. (2014). H3S28 phosphorylation is a hallmark of the transcriptional response to cellular stress. Genome Res. 24, 1808–1820. doi: 10.1101/gr.176255.114, PMID: 25135956 PMC4216922

[ref185] Sawicka A. Seiser C. (2012). Histone H3 phosphorylation - a versatile chromatin modification for different occasions. Biochimie 94, 2193–2201. doi: 10.1016/j.biochi.2012.04.018, PMID: 22564826 PMC3480636

[ref186] Schmitz S. U. Grote P. Herrmann B. G. (2016). Mechanisms of long noncoding RNA function in development and disease. Cell. Mol. Life Sci. 73, 2491–2509. doi: 10.1007/s00018-016-2174-5, PMID: 27007508 PMC4894931

[ref187] Sekar D. Tusubira D. Ross K. (2022). TDP-43 and NEAT long non-coding RNA: roles in neurodegenerative disease. Front. Cell. Neurosci. 16:954912. doi: 10.3389/fncel.2022.954912, PMID: 36385948 PMC9650703

[ref188] Shahriari M. Kesharwani P. Johnston T. P. Sahebkar A. (2023). Anticancer potential of curcumin-cyclodextrin complexes and their pharmacokinetic properties. Int. J. Pharm. 631:122474. doi: 10.1016/j.ijpharm.2022.122474, PMID: 36509227

[ref189] Sharma N. Nehru B. (2018). Curcumin affords neuroprotection and inhibits α-synuclein aggregation in lipopolysaccharide-induced Parkinson’s disease model. Inflammopharmacology 26, 349–360. doi: 10.1007/s10787-017-0402-8, PMID: 29027056

[ref190] Shi Q. Yang X. (2016). Circulating MicroRNA and Long noncoding RNA as biomarkers of cardiovascular diseases. J. Cell. Physiol. 231, 751–755. doi: 10.1002/jcp.25174, PMID: 26308238

[ref191] Shimizu K. Sunagawa Y. Funamoto M. Wakabayashi H. Genpei M. Miyazaki Y.. (2020). The synthetic curcumin analogue GO-Y030 effectively suppresses the development of pressure overload-induced heart failure in mice. Sci. Rep. 10:7172. doi: 10.1038/s41598-020-64207-w, PMID: 32346115 PMC7188884

[ref192] Shoba G. Joy D. Joseph T. Majeed M. Rajendran R. Srinivas P. S. (1998). Influence of piperine on the pharmacokinetics of curcumin in animals and human volunteers. Planta Med. 64, 353–356. doi: 10.1055/s-2006-957450, PMID: 9619120

[ref193] Shojaei M. Foshati S. Abdi M. Askari G. Sukhorukov V. N. Bagherniya M.. (2023). The effectiveness of nano-curcumin on patients with COVID-19: a systematic review of clinical trials. Phytother. Res. 37, 1663–1677. doi: 10.1002/ptr.7778, PMID: 36799442

[ref194] Shukla P. K. Khanna V. K. Khan M. Y. Srimal R. C. (2003). Protective effect of curcumin against lead neurotoxicity in rat. Hum. Exp. Toxicol. 22, 653–658. doi: 10.1191/0960327103ht411oa, PMID: 14992327

[ref195] Smith T. J. Ashar B. H. (2019). Iron deficiency anemia due to high-dose turmeric. Cureus 11:e3858. doi: 10.7759/cureus.3858, PMID: 30899609 PMC6414192

[ref196] St Laurent G. Wahlestedt C. Kapranov P. (2015). The landscape of long noncoding RNA classification. Trends Genet. 31, 239–251. doi: 10.1016/j.tig.2015.03.007, PMID: 25869999 PMC4417002

[ref197] Stohs S. J. Chen O. Ray S. D. Ji J. Bucci L. R. Preuss H. G. (2020). Highly bioavailable forms of curcumin and promising avenues for curcumin-based research and application: a review. Molecules 25:397. doi: 10.3390/molecules25061397, PMID: 32204372 PMC7144558

[ref198] Strimpakos A. S. Sharma R. A. (2008). Curcumin: preventive and therapeutic properties in laboratory studies and clinical trials. Antioxid. Redox Signal. 10, 511–546. doi: 10.1089/ars.2007.1769, PMID: 18370854

[ref199] Sunagawa Y. Funamoto M. Shimizu K. Shimizu S. Sari N. Katanasaka Y.. (2021). Curcumin, an inhibitor of p300-HAT activity, suppresses the development of hypertension-induced left ventricular hypertrophy with preserved ejection fraction in dahl rats. Nutrients 13:608. doi: 10.3390/nu13082608, PMID: 34444769 PMC8397934

[ref200] Swygert S. G. Peterson C. L. (2014). Chromatin dynamics: interplay between remodeling enzymes and histone modifications. Biochim. Biophys. Acta 1839, 728–736. doi: 10.1016/j.bbagrm.2014.02.013, PMID: 24583555 PMC4099280

[ref201] Szymusiak M. Hu X. Plata P. A. L. Ciupinski P. Wang Z. J. Liu Y. (2016). Bioavailability of curcumin and curcumin glucuronide in the central nervous system of mice after oral delivery of nano-curcumin. Int. J. Pharm. 511, 415–423. doi: 10.1016/j.ijpharm.2016.07.027, PMID: 27426105

[ref202] Tajima S. Suetake I. Takeshita K. Nakagawa A. Kimura H. Song J. (2022). Domain structure of the Dnmt1, Dnmt3a, and Dnmt3b DNA methyltransferases. Adv. Exp. Med. Biol. 1389, 45–68. doi: 10.1007/978-3-031-11454-0_3, PMID: 36350506 PMC11025882

[ref203] Tan C. Zhou L. Wen W. Xiao N. (2021). Curcumin promotes cholesterol efflux by regulating ABCA1 expression through miR-125a-5p/SIRT6 axis in THP-1 macrophage to prevent atherosclerosis. J. Toxicol. Sci. 46, 209–222. doi: 10.2131/jts.46.209 33952798

[ref204] Tang X. Luo Y. Yuan D. Calandrelli R. Malhi N. K. Sriram K.. (2023). Long noncoding RNA LEENE promotes angiogenesis and ischemic recovery in diabetes models. J. Clin. Invest. 133:759. doi: 10.1172/JCI161759, PMID: 36512424 PMC9888385

[ref205] Tikoo K. Meena R. L. Kabra D. G. Gaikwad A. B. (2008). Change in post-translational modifications of histone H3, heat-shock protein-27 and MAP kinase p38 expression by curcumin in streptozotocin-induced type I diabetic nephropathy. Br. J. Pharmacol. 153, 1225–1231. doi: 10.1038/sj.bjp.0707666, PMID: 18204486 PMC2275435

[ref206] Tønnesen H. H. Másson M. Loftsson T. (2002). Studies of curcumin and curcuminoids. XXVII. Cyclodextrin complexation: solubility, chemical and photochemical stability. Int. J. Pharm. 244, 127–135. doi: 10.1016/S0378-5173(02)00323-X, PMID: 12204572

[ref207] Urrutia G. De Assuncao T. M. Mathison A. J. Salmonson A. Kerketta R. Zeighami A.. (2021). Inactivation of the euchromatic histone-lysine N-methyltransferase 2 pathway in pancreatic epithelial cells antagonizes cancer initiation and pancreatitis-associated promotion by altering growth and immune gene expression networks. Front. Cell Dev. Biol. 9:681153. doi: 10.3389/fcell.2021.681153, PMID: 34249932 PMC8261250

[ref208] Vallée A. Lecarpentier Y. (2020). Curcumin and endometriosis. Int. J. Mol. Sci. 21:440. doi: 10.3390/ijms21072440, PMID: 32244563 PMC7177778

[ref209] Vavouri T. Lehner B. (2012). Human genes with CpG island promoters have a distinct transcription-associated chromatin organization. Genome Biol. 13, 1–12. doi: 10.1186/gb-2012-13-11-r110, PMID: 23186133 PMC3580500

[ref210] Vogel H. Pelletier J. (1815). Curcumin-biological and medicinal properties. J. Pharma. 2, 24–29.

[ref211] Wada T. T. Araki Y. Sato K. Aizaki Y. Yokota K. Kim Y. T.. (2014). Aberrant histone acetylation contributes to elevated interleukin-6 production in rheumatoid arthritis synovial fibroblasts. Biochem. Biophys. Res. Commun. 444, 682–686. doi: 10.1016/j.bbrc.2014.01.195, PMID: 24513290

[ref212] Wan Mohd Tajuddin W. N. B. Lajis N. H. Abas F. Othman I. Naidu R. (2019). Mechanistic understanding of curcumin's therapeutic effects in lung Cancer. Nutrients 11:989. doi: 10.3390/nu11122989, PMID: 31817718 PMC6950067

[ref213] Wang Y. Wang Y. Luo M. Wu H. Kong L. Xin Y.. (2015). Novel curcumin analog C66 prevents diabetic nephropathy via JNK pathway with the involvement of p300/CBP-mediated histone acetylation. Biochim. Biophys. Acta 1852, 34–46. doi: 10.1016/j.bbadis.2014.11.006, PMID: 25446993 PMC4369325

[ref214] Wang Q. Xiong F. Wu G. Liu W. Chen J. Wang B.. (2022). Gene body methylation in cancer: molecular mechanisms and clinical applications. Clin. Epigenetics 14:154. doi: 10.1186/s13148-022-01382-9, PMID: 36443876 PMC9706891

[ref215] Wang Y. Zhang H. Hua L. Wang Z. Geng S. Zhang H.. (2022). Curcumin prevents Alzheimer's disease progression by upregulating JMJD3. Am. J. Transl. Res. 14, 5280–5294. 36105064 PMC9452350

[ref216] Wang X. Zhang S. Li Y. Zhang Y. (2025). The regulation of miRNAs using curcumin and other polyphenols during the prevention and treatment of Alzheimer's disease. Hum. Mol. Genet. 34, 117–127. doi: 10.1093/hmg/ddae154, PMID: 39561994

[ref217] Wanninger S. Lorenz V. Subhan A. Edelmann F. T. (2015). Metal complexes of curcumin--synthetic strategies, structures and medicinal applications. Chem. Soc. Rev. 44, 4986–5002. doi: 10.1039/c5cs00088b, PMID: 25964104

[ref218] Wu J. Hu J. Zhang F. Jin Q. Sun X. (2022). High glucose promotes IL-17A-induced gene expression through histone acetylation in retinal pigment epithelium cells. Int. Immunopharmacol. 110:108893. doi: 10.1016/j.intimp.2022.108893, PMID: 35978498

[ref219] Wu J. Wu S. Shi L. Zhang S. Ren J. Yao S.. (2017). Design, synthesis, and evaluation of asymmetric EF24 analogues as potential anti-cancer agents for lung cancer. Eur. J. Med. Chem. 125, 1321–1331. doi: 10.1016/j.ejmech.2016.10.027, PMID: 27886548

[ref220] Xiao J. Cai X. Zhou W. Wang R. Ye Z. (2022). Curcumin relieved the rheumatoid arthritis progression via modulating the linc00052/miR-126-5p/PIAS2 axis. Bioengineered. 13, 10973–10983. doi: 10.1080/21655979.2022.2066760, PMID: 35473503 PMC9208441

[ref221] Xie L. Ji X. Zhang Q. Wei Y. (2022). Curcumin combined with photodynamic therapy, promising therapies for the treatment of cancer. Biomed. Pharmacother. 146:112567. doi: 10.1016/j.biopha.2021.112567, PMID: 34953392

[ref222] Xu H. Nie B. Liu L. Zhang C. Zhang Z. Xu M.. (2019). Curcumin prevents brain damage and cognitive dysfunction during ischemic-reperfusion through the regulation of miR-7-5p. Curr. Neurovasc. Res. 16, 441–454. doi: 10.2174/1567202616666191029113633, PMID: 31660818

[ref223] Xu Y. Sun H. Lv J. Wang Y. Zhang Y. Wang F. (2023). Effects of polysaccharide thickening agent on the preparation of walnut oil oleogels based on methylcellulose: characterization and delivery of curcumin. Int. J. Biol. Macromol. 232:123291. doi: 10.1016/j.ijbiomac.2023.123291, PMID: 36652980

[ref224] Yadav A. Lomash V. Samim M. Flora S. J. (2012). Curcumin encapsulated in chitosan nanoparticles: a novel strategy for the treatment of arsenic toxicity. Chem. Biol. Interact. 199, 49–61. doi: 10.1016/j.cbi.2012.05.011, PMID: 22704994

[ref225] Yallapu M. M. Nagesh P. K. B. Jaggi M. Chauhan S. C. (2015). Therapeutic applications of curcumin nanoformulations. AAPS J. 17, 1341–1356. doi: 10.1208/s12248-015-9811-z, PMID: 26335307 PMC4627456

[ref226] Yan C. Zhang Y. Zhang X. Aa J. Wang G. Xie Y. (2018). Curcumin regulates endogenous and exogenous metabolism via Nrf2-FXR-LXR pathway in NAFLD mice. Biomed. Pharmacother. 105, 274–281. doi: 10.1016/j.biopha.2018.05.135, PMID: 29860219

[ref227] Yang H. Li K. Wang T. Zou Y. Xu G. Zhou C.. (2025). Novel dual-functional half-curcumin analogues as a fluorescent and PET probe for β-amyloid imaging in the Alzheimer's disease APP/PS1 model. ACS Chem. Neurosci. 16, 365–373. doi: 10.1021/acschemneuro.4c00532, PMID: 39818695

[ref228] Yu B. Xie C. Yu S. Hu Y. (2021). Long noncoding RNA and atrial fibrillation. Zhong Nan Da Xue Xue Bao Yi Xue Ban 46, 877–883. doi: 10.11817/j.issn.1672-7347.2021.200531, PMID: 34565733 PMC10929980

[ref229] Yun J. M. Jialal I. Devaraj S. (2011). Epigenetic regulation of high glucose-induced proinflammatory cytokine production in monocytes by curcumin. J. Nutr. Biochem. 22, 450–458. doi: 10.1016/j.jnutbio.2010.03.014, PMID: 20655188 PMC3010508

[ref230] Zhang M. M. Bahal R. Rasmussen T. P. Manautou J. E. Zhong X. B. (2021). The growth of siRNA-based therapeutics: updated clinical studies. Biochem. Pharmacol. 189:114432. doi: 10.1016/j.bcp.2021.114432, PMID: 33513339 PMC8187268

[ref231] Zhang T. Chen X. Qu Y. Ding Y. (2021). Curcumin alleviates oxygen-glucose-deprivation/reperfusion-induced oxidative damage by regulating miR-1287-5p/LONP2 Axis in SH-SY5Y cells. Anal. Cell. Pathol. 2021, 1–12. doi: 10.1155/2021/5548706, PMID: 34589382 PMC8476263

[ref232] Zhang L. Lu Q. Chang C. (2020). Epigenetics in health and disease. In: C. Chang, Q. Lu, (eds) Epigenetics in Allergy and Autoimmunity. Advances in Experimental Medicine and Biology, Singapore: Springer. 1253. doi: 10.1007/978-981-15-3449-2_1, PMID: 32445090

[ref233] Zhang J. Wang Q. Rao G. Qiu J. He R. (2019). Curcumin improves perfusion recovery in experimental peripheral arterial disease by upregulating microRNA-93 expression. Exp. Ther. Med. 17, 798–802. doi: 10.3892/etm.2018.7000, PMID: 30651865 PMC6307414

[ref234] Zhang P. Wu W. Chen Q. Chen M. (2019). Non-coding RNAs and their integrated networks. J. Integr. Bioinform. 16:27. doi: 10.1515/jib-2019-0027, PMID: 31301674 PMC6798851

[ref235] Zhang J. Ye Y. Xu Z. Luo M. Wu C. Zhang Y.. (2023). Histone methyltransferase KMT2D promotes prostate cancer progression through paracrine IL-6 signaling. Biochem. Biophys. Res. Commun. 655, 35–43. doi: 10.1016/j.bbrc.2023.02.083, PMID: 36924677

[ref236] Zhao H. Wang L. Zhang L. Zhao H. (2023). Phytochemicals targeting lncRNAs: a novel direction for neuroprotection in neurological disorders. Biomed. Pharmacother. 162:114692. doi: 10.1016/j.biopha.2023.114692, PMID: 37058817

[ref237] Zhou F. Hu X. Feng W. Li M. Yu B. Fu C.. (2021). LncRNA H19 abrogates the protective effects of curcumin on rat carotid balloon injury via activating Wnt/β-catenin signaling pathway. Eur. J. Pharmacol. 910:174485. doi: 10.1016/j.ejphar.2021.174485, PMID: 34487706

[ref238] Zhou S. Zhang S. Shen H. Chen W. Xu H. Chen X.. (2017). Curcumin inhibits cancer progression through regulating expression of microRNAs. Tumour Biol. 39:1010428317691680. doi: 10.1177/1010428317691680, PMID: 28222667

[ref239] Zhu L. Yang M. Fan L. Yan Q. Zhang L. Mu P.. (2025). Interaction between resveratrol and SIRT1: role in neurodegenerative diseases. Naunyn Schmiedeberg's Arch. Pharmacol. 398, 89–101. doi: 10.1007/s00210-024-03319-w, PMID: 39105797

[ref240] Zia A. Farkhondeh T. Pourbagher-Shahri A. M. Samarghandian S. (2021). The role of curcumin in aging and senescence: molecular mechanisms. Biomed. Pharmacother. 134:111119. doi: 10.1016/j.biopha.2020.111119 33360051

